# Analgesic Efficacy of Adjuvant Medications in the Pediatric Caudal Block for Infraumbilical Surgery: A Network Meta-Analysis of Randomized Controlled Trials

**DOI:** 10.7759/cureus.28582

**Published:** 2022-08-30

**Authors:** Ushma J Shah, Niveditha Karuppiah, Hovhannes Karapetyan, Janet Martin, Herman Sehmbi

**Affiliations:** 1 Anesthesia & Perioperative Medicine, London Health Sciences Centre, Western University, London, CAN; 2 Surgery, Yerevan State Medical University, Yerevan, ARM

**Keywords:** network meta-analysis, pain, post-operative, adjuvant, caudal, local anesthesia, pediatric

## Abstract

Various adjuvants are added to local anesthetics in caudal block to improve analgesia. The comparative analgesic effectiveness and relative rankings of these adjuvants are unknown.

This network meta-analysis (NMA) sought to evaluate the comparative analgesic efficacy and relative ranking of caudal adjuvants added to local anesthetics (versus local anesthetics alone) in pediatric infra-umbilical surgery. We searched the United States National Library of Medicine database (MEDLINE), PubMed, and Excerpta Medica database (Embase) for randomized controlled trials (RCTs) comparing caudal adjuvants (clonidine, dexmedetomidine, ketamine, magnesium, morphine, fentanyl, tramadol, dexamethasone, and neostigmine) among themselves, or to no adjuvant (control). We performed a frequentist NMA and employed Cochrane’s ‘Risk of Bias’ tool to evaluate study quality. We chose the duration of analgesia (defined as 'the time from caudal injection to the time of rescue analgesia') as our primary outcome. We also assessed the number of analgesic dose administrations and total dose of acetaminophen within 24 h.

The duration of analgesia [87 randomized control trials (RCTs), 5285 patients] was most prolonged by neostigmine [mean difference: 513 min, (95% confidence interval, CI: 402, 625)]. Dexmedetomidine reduced the frequency of analgesic dose administrations within 24 h [29 RCTs, 1765 patients; -1.2 dose (95% CI: -1.6, -0.9)] and the total dose of acetaminophen within 24 h [18 RCTs, 1156 patients; -350 mg (95% CI: -467, -232)] the most.

Among caudal adjuvants, neostigmine (moderate certainty), tramadol (low certainty), and dexmedetomidine (low certainty) prolonged the duration of analgesia the most. Dexmedetomidine also reduced the analgesic frequency and consumption more than other caudal adjuvants (moderate certainty).

## Introduction and background

Introduction

A caudal epidural block is a common regional analgesic technique in pediatric surgery [1]. It is a time-tested, safe, and efficacious technique [2]. However, the duration of post-operative pain seen with much pediatric surgery (>24 h) outlasts the duration of analgesia afforded by a standard 'local-anesthetics only' caudal block (4-12 h) [3]. While continuous catheters prolong analgesic duration, such techniques are more cumbersome, require significant technical expertise [4], and may be associated with higher adverse events. Contrary to this, adding adjuvants to local anesthetic is an appealing alternative. Adjuvants can improve the block and analgesic duration [5], reduce general anesthetic [6] or local anesthetic requirements [7], allow for smoother emergence, lower incidence of emergence delirium [8], and facilitate early discharge in ambulatory surgery.

Various adjuvants have been shown to enhance caudal blocks with varying degrees of success. A multitude of clinical trials and meta-analyses have analyzed the efficacy of different adjuvants such as alpha-2 agonists (clonidine [9] and dexmedetomidine [8]), N-methyl-D-aspartate (NMDA) agonists (ketamine [10] and magnesium [11]), opioids (fentanyl, morphine, and tramadol [12]), corticosteroids (dexamethasone [13-14]), and acetylcholine esterase inhibitors (neostigmine) [12]. The European Society of Regional Anesthesia and Pain Therapy (ESRA) and the American Society of Regional Anesthesia and Pain Medicine (ASRA) joint committee practice advisory on pediatric regional anesthesia [3] provides specific recommendations on many adjuvants but given a plethora of recent studies; this advisory is likely already outdated. Furthermore, while each adjuvant is superior to the control (no adjuvant), it is difficult to ascertain the most efficacious agent (or their comparative rankings) based on clinical trials or meta-analyses alone. Network meta-analysis (NMA) represents a methodology that can qualitatively and quantitatively assess the overall evidence and provide comparative rankings of caudal adjuvants across multiple outcomes. Compared to conventional pairwise meta-analysis, NMA identifies findings often and earlier [15]. Therefore, such a review would inform the advisory and clinical practice. 

In this systematic review and NMA of randomized controlled trials (RCTs), we sought the relative extent to which adjuvants enhance the efficacy of caudal block in pediatric patients undergoing infraumbilical surgery. Specifically, we aimed to rank the comparative effectiveness of different adjuvants on the duration of analgesia, the number of analgesic dose administrations, and the total dose of acetaminophen within 24 h post-operatively.

## Review

Methods

Protocol and Registration

We prospectively registered a protocol for this NMA (PROSPERO, CRD42018108345). After submission, no methodological changes were made to the protocol (Section 1, Appendix). In preparing this manuscript, we adhered to the Preferred Reporting Items for Systematic Reviews and Meta-Analyses extension statement for reporting systematic reviews incorporating NMAs of health care interventions (PRISMA-NMA) [16]. No institutional approval was needed, given that this review analyses previously published data. 

Eligibility Criteria

We sought RCTs of pediatric patients undergoing infra-umbilical surgery under caudal epidural blocks (under a general anesthetic or sedation). The RCTs must compare the caudal route of nine adjuvants (clonidine, dexmedetomidine, ketamine, magnesium, morphine, fentanyl, tramadol, dexamethasone, and neostigmine) among themselves or no adjuvant (control). RCTs should have used long-acting local anesthetics (bupivacaine, levobupivacaine, or ropivacaine) and performed using landmark technique or ultrasound guidance. We did not exclude RCTs employing lidocaine or epinephrine to accelerate the block onset. RCTs should have assessed outcomes about analgesic efficacy -- the duration of analgesia, the number of analgesic dose administrations, and the total dose of acetaminophen within 24 h post-operatively. Finally, only RCTs are indexed in major databases, published in English, and available in full text. We excluded studies if they were RCTs involving adult patients (age > 18 years); animal, volunteer, or cadaveric studies; supra-umbilical surgery; and the predominant use of short-acting local anesthetic agents. We excluded unpublished studies, conference proceedings, thesis, and abstracts.

Information Sources and Search Strategy

An information specialist searched three databases: the US National Library of Medicine (MEDLINE), PubMed, and Excerpta Medica (Embase). We used medical subject headings (MeSH), text words, and controlled vocabulary terms relating to 'clonidine,' 'dexmedetomidine,' 'ketamine,' 'magnesium,' 'morphine,' 'fentanyl,' 'tramadol,' 'dexamethasone,' and 'neostigmine,' 'caudal epidural block,' 'local anesthesia,' and 'randomized controlled trial.' The search was limited to human RCTs published in English between 1946 and June 2020. Section 2 in the Appendix summarizes the search strategy.

Study Selection

Two authors (N.K and U.S) independently evaluated the retrieved abstracts and applied eligibility criteria to include or exclude retrieved studies. A third author (H.S) mediated consensus to resolve disagreements (if any).

Data Collection Process

Two authors (N.K and H.K) independently (and induplicate) extracted relevant study characteristics and outcome data. We resolved any disagreements by consulting with a third author (H.S). We did not contact the authors for original data due to a large number of studies. We collected the following data using Microsoft Excel (Microsoft Corp, Redmond, WA, USA): study characteristics -- first author name, year of publication, study title, journal name, country of study, type of surgery, type of intraoperative anesthesia used, and details of the groups with the number of patients in each group; block characteristics and analgesic regimens -- local anesthetic details (type, volume, and concentration), dose of adjuvant, block localization technique (ultrasound, peripheral nerve stimulation or landmark guided), use of any intra-operative rescue drugs, and perioperative analgesia regimen [preoperative, intraoperative, post-anesthesia care unit (PACU), and post-operative]; and analgesic outcomes -- the duration of analgesia, number of analgesic dose administration within 24 h, and total dose of acetaminophen within 24 h.

We chose the duration of analgesia (defined as 'the time from caudal injection to the time of rescue analgesia') as our primary outcome. Most RCTs employ a threshold of pain score to trigger the provision of rescue analgesics. If such a threshold was not specified, but the duration of analgesia provided, we extracted such published outcome data for analysis. To assess homogeneity, we extracted each paper's study-specific definitions of the primary outcome. We designated all number of analgesic dose administration required (within 24 h) and total dose of acetaminophen (within 24 h) as secondary outcomes. 

Network Geometry

We constructed a network map of intervention with different caudal adjuvants representing each treatment node and the control (no adjuvant) representing the common comparator for each outcome. We pooled different doses of the same adjuvant as this meant to preserve the network geometry. If an RCT compared multiple doses of the same adjuvant to control, we used data from the arm employing the smallest dose of the adjuvant (and control arm). We dropped arms in RCTs comparing caudal adjuvants via non-neuraxial (e.g., intravenous) routes from the analysis. The resulting networks informed assessments of feasibility and consistency. 

Risk of Bias 

Two authors (H.S and N.K) independently assessed the methodological quality of included RCTs using the Cochrane Collaboration Risk of Bias tool (version 2, 2016) for RCTs [17]. This quality appraisal tool evaluates RCTs for biases, including randomization process (random sequence generation, allocation concealment, and baseline imbalances); deviation from intended interventions (blinding of participants and personnel, treatment adherence, balanced co-interventions, the success of treatment allocation); missing outcome data (significant or differential missing data or loss to follow-up); measurement of outcome (blinding of outcome assessors, use of subjective outcomes); and selection of reported results (selective or partial reporting of data or analysis). The authors assigned a score (low, some concern, or high risk of bias) to each type of bias category, with the highest bias rating representing the overall bias rating. Multiple domains with some concerns also yielded an overall rating of high risk of bias. The risk of bias was evaluated for each outcome, of each study. Additionally, we constructed contribution-specific risk of bias across each comparison arm (e.g., dexmedetomidine vs. clonidine) [18]. We resolved disagreements by consulting with a third author (U.J). Studies were not excluded based on their respective risk of bias.

Summary Measures

We extracted continuous data as mean and standard deviation (SD). When median and range were available, these estimates were derived using the method described by Hozo et al. [19] and Wan et al. [20]. We used simple imputations to impute SDs when not reported [21]. For continuous outcome, we used the weighted mean difference (WMD) with 95% confidence intervals (CI) to measure the difference in effect size between each pairwise comparison. We interpreted the potential differences in results between groups in the context of a minimal clinically important difference (MCID) of 25% of the effect size of outcomes in the control groups for each outcome. We identified this as 100 min for the analgesic duration, 0.5 doses for the number of dose administration, and 120 mg of acetaminophen for the analgesic dose. We arrived at this definition of MCID through discussion and consensus among the local intra-department clinicians. We have described our detailed statistical methods in the Section 2 of the Appendix.

Statistical Analysis

We used the R-statistical package (R Studio v 1.4.1) for frequentist statistical analysis (netmeta package [22]). We also employed frequentist methods using STATA v 14.0 (StataCorp, USA; network package [23-24]) and Bayesian methods in R Studio (BUGSnet package [25]). The details on the use of multiple packages (with reasons) are provided in the appendix. Two authors (H.S and U.S) performed the statistical analysis and checked for errors by the third (JM). We conducted a pairwise frequentist meta-analysis using the DerSimonian Laird random-effects model [26]. We considered differences statistically significant if p < 0.05 (two-sided) or when values of 0 and 1 were not included in the 95% CI for continuous and dichotomous outcomes, respectively. We used the I2 statistic to identify statistical heterogeneity [27]. We employed contrast-based parametrization [28], data augmentation, and assumed common heterogeneity variance across all pairwise comparisons. We assessed network geometry, assigning the node size that reflects the corresponding sample size and arm width that reflects the corresponding number of studies [29]. We obtained the resultant mixed (or network) estimates assuming the consistency model (i.e., heterogeneity is independent of the comparison examined) and constructed league tables of mixed estimates for each outcome. We assessed each network's global inconsistency (frequentist and Bayesian) and local inconsistency. Using the contribution matrix, we analyzed the contribution of each mixed estimate's direct vs. indirect comparisons [18]. We produced a ranking of the adjuncts for each outcome of interest using the surface under the cumulative ranking curve (SUCRA) [23], yielding a probability (percentage) of an intervention being among the best options and a mean rank. Finally, we combined results from all analgesic outcomes to ascertain the best adjuvant across all analgesic outcomes using a 'rank-heat plot’ [30].

Assessment of Inconsistency

Inconsistency may invalidate the findings of an NMA. We evaluated inconsistency between the direct and indirect estimates using the global approach in both frequentists (design-by-treatment model, Higgins and co-workers [31]) and the Bayesian framework (leverage plot [25]). We also visually inspected the network forest plots to assess agreements between the consistency and inconsistency models in the frequentist method (Wald test) as well as Bayesian methods (DIC and model performance). We investigated local inconsistencies using node-splitting [32]. We planned to present results as mixed estimates if global inconsistency was not detected. We downgraded the evidence if we identified significant local inconsistencies.

Publication Bias

We evaluated statistical evidence of publication bias for each outcome for pairwise comparisons by visually inspecting Begg's funnel plot for asymmetry and conducting an Egger's regression test [33]. At the network level, publication bias was assessed using a 'comparison-adjusted' funnel plot’ [34]. This depicts the difference between the study-specific effect sizes from the corresponding comparison-specific summary effect for each comparison in a network and plots this on the horizontal axis. The 'comparison-adjusted' funnel plot should be symmetric around the zero line without small-study effects.

Additional Analysis

We recognized that clinical and methodological differences between studies potentially introduce significant statistical heterogeneity. Thus, we planned to explore this heterogeneity using subgroups analysis (risk of bias and type of local anesthetic) and meta-regression analysis (local anesthetic volume and concentration; adjuvant dose). We performed such network meta-regression using a Bayesian framework (frequentist package 'netmeta' in R is unable to do so). We anticipated only a few studies to use lidocaine or epinephrine. Thus we did not study a formal analysis of the use of such agents, as it would likely lead to disconnected networks.

Grading of Recommendations

We assessed the certainty of evidence from the NMA results using the GRADE approach [35,36] using CINeMa platform and methodology [18]. Such an assessment differs from the pairwise meta-analyses in critical aspects. Six domains that affect confidence in the NMA results are within-study bias, reporting bias, indirectness, imprecision, heterogeneity, and incoherence (or inconsistency). In this way, reviewers assess the level of concerns for each relative treatment effect from NMA as giving rise to 'no concerns,' 'some concerns,' or 'major concerns' in each of the six domains. Finally, we summarized judgments across the domains into a single confidence rating ('high,' 'moderate,' 'low,' or 'very low').

Results

Study Selection

**Figure 1:**
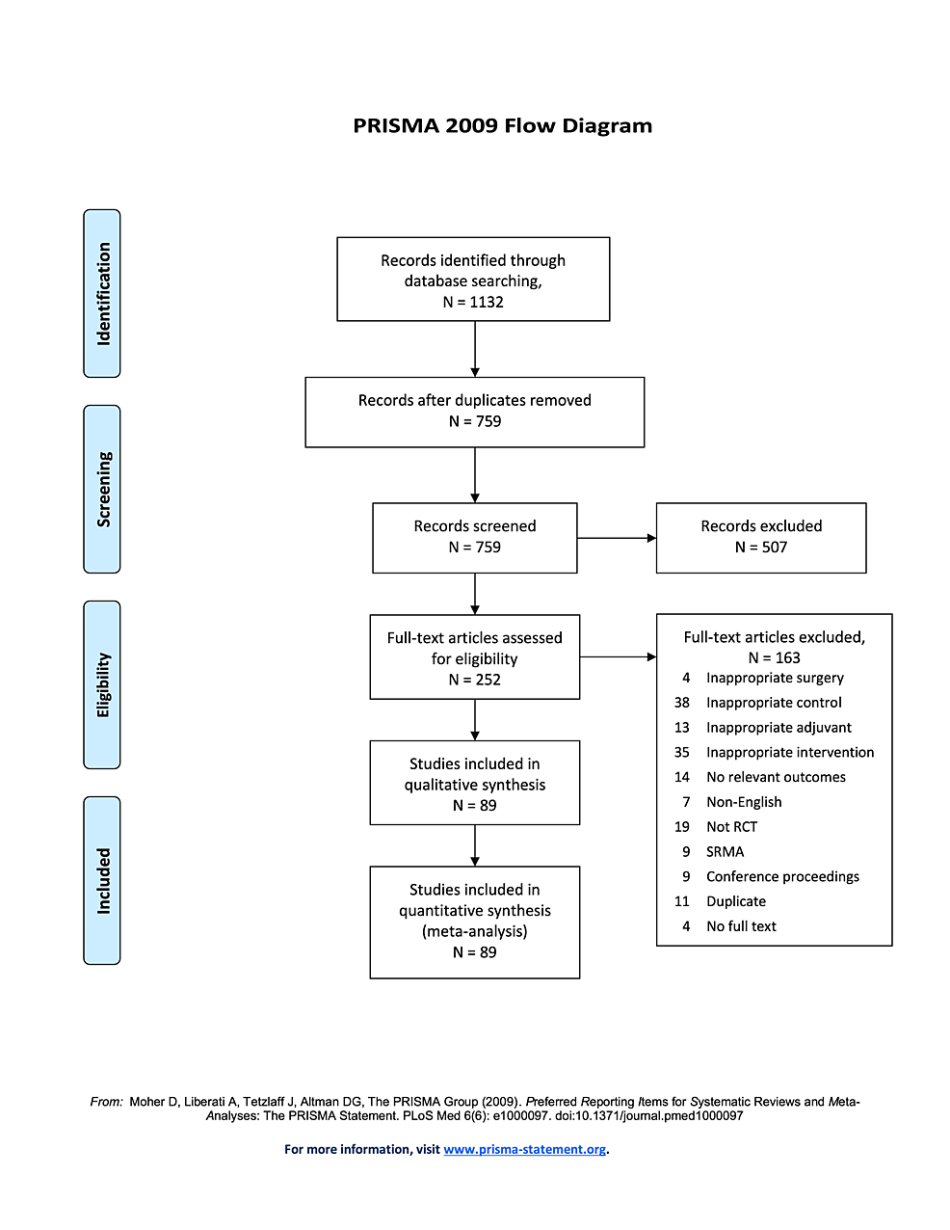
PRISMA flow diagram of study inclusion and exclusion. PRISMA, Preferred Reporting Items for Systematic Reviews and Meta-analyses.

Our search identified 1132 records, which yielded 759 records after de-duplication. Of these, we screened 252 full-text records for eligibility. Finally, we included 89 unique records in this review. This screening process is summarized in Figure 1 (PRISMA flow diagram) [16].

Study Characteristics

The RCTs ranged from 1995 to 2019, with a majority (63 studies) conducted recently, from 2010 to 2019. Most studies originated in India (n=42), followed by Egypt (n=11) and Turkey (n=10). Most studies were published in Pediatric Anesthesia (n=9), followed by the *Indian Journal of Anesthesia* (n=7) and *Journal of Anesthesiology & Clinical Pharmacology* (n=7). Most patients were healthy with ASA class I (n=23) or I-II (n=62). Thirty-five RCTs included patients aged six years or younger, while 52 RCTs also included six or older patients. Most RCTs employed a general anesthetic (n=85) to allow the surgery and used landmark technique to guide the caudal block (n=83). Bupivacaine was used in 58 RCTs, Ropivacaine in 21 RCTs, and Levobupivacaine in 10 RCTs. Most studies employed a concentration of 0.25% (n=58) and a volume-based dosing of 1 mL/kg (n=56) for the block. FLACC [37] (Face, Legs, Activity, Crying & Consolability Scale; n=30), CHIPPS [38] (Children & Infants Postoperative Pain; n=10), and MOPS [39] (Modified Objective Pain Scale n=7) were the most commonly employed pain scales for pain management. Finally, all included RCTs were prospective clinical trials, employing a parallel two-arm (n=70), three-arm (n=15), or a four-arm (n=4) design. Despite these differences, most studies employed common methods, including the definition of the primary outcome and assessment methods. These common methods assured us of transitivity in this NMA. We have summarized the general characteristics (Table 1), the analgesic regimen (Table 2), the outcome characteristics (Table 3), and the overall summary of included studies (Table 4) below.

**Table 1:** General characteristics of RCTs included in the review. RCT, randomized control trial;  ASA Class, American Society of Anesthesiology classification; GA, general anesthesia; mL/kg, milliliters per kilograms; mcg/kg, microgram per kilogram; mg/kg, milligram per kilogram; mg, milligram #, lidocaine used; $, epinephrine used

Name, Year, Country	Journal	ASA class, age, anesthetic, guidance	Surgery	Local anesthetic concentration & volume	Group 1	Group 2	Group 3	Group 4	Sample size
Abu-Elyazed (2017) Egypt [40]	Egyptian Journal of Anesthesia	I-II, 1-6 years, GA, Ultrasound	Inguinal hernia	0.25% Bupivacaine 0.75 ml/kg	Control (no adjuvant)	Dexamethasone 0.1 mg/kg	Neostigmine 2 mcg/kg		35/35/35
Ahuja (2014) India [41]	Journal of Anesthesiology Clinical Pharmacology	I-II, 2-10 years, GA, Landmark	Inguinal hernia, circumcision, hypospadias	0.25% Bupivacaine 1 ml/kg	Control (no adjuvant)	Fentanyl 1 mcg/kg	Ketamine 0.5 mg/kg		20/20/20
Ahuja (2015) India [42]	Journal of Clinical & Diagnostic Research	I-II, 1-10 years, GA, Landmark	Inguinal hernia, circumcision, hypospadias	0.25% Bupivacaine 1 ml/kg	Control (no adjuvant)	Fentanyl 1 mcg/kg	Clonidine 3 mcg/kg		20/20/20
Akin (2010) Turkey [43]	Pediatric Anesthesia	I-II, 2-8 years, GA, Landmark	Inguinal hernia, orchidopexy	0.25% Levobupivacaine 0.75 ml/kg	Control (no adjuvant)	Clonidine 2 mcg/kg			30/30
Al-Zaben (2015) Jordan [44]	Pediatric Anesthesia	I, 1-6 years, GA, Landmark	Inguinal hernia, orchidopexy, circumcision, hydrocele, hypospadias	0.25% Bupivacaine 0.8 ml/kg	Control (no adjuvant)	Dexmedetomidine 1 mcg/kg			29/29
Al-Zaben (2016) Jordan [45]	Journal of Clinical Anesthesia	I, 1-6 years, GA, Landmark	Inguinal hernia, orchidopexy, circumcision, hydrocele, hypospadias	0.25% Bupivacaine 1 ml/kg	Control (no adjuvant)	Dexmedetomidine 1 mcg/kg			30/31
Aliena (2018) India [46]	Indian Journal of Anesthesia	I-II, 1-12 years, GA, Landmark	Inguinal hernia, orchidopexy, hypospadias	0.25% Bupivacaine 0.75 ml/kg	Control (no adjuvant)	Ketamine 0.5 mg/kg			30/30
Amitha (2019) India [47]	Anesthesia Essays & Research	I-II, 2-12 years, GA, Landmark	Inguinal hernia, orchidopexy, circumcision, hypospadias, other infra-inguinal procedures	0.25% Bupivacaine 0.5 ml/kg	Clonidine 2mcg/kg	Tramadol 2mcg/kg			30/30
Anand (2011) India [48]	Indian Journal of Anesthesia	I-II, 6 months - 6 years, GA, Landmark	Inguinal hernia, circumcision, urethroplasty, other urological procedures	0.25% Bupivacaine 1 ml/kg	Control (no adjuvant)	Dexmedetomidine 2 mcg/kg			21/21/21
Aziz (2016) Egypt [49]	Ain-Shams Journal of Anesthesiology	I-II, 1-5 years, GA, USG	Inguinal hernia, orchidopexy, circumcision	0.25% Levobupivacaine 0.7 ml/kg	Control (no adjuvant)	Dexmedetomidine 1 mcg/kg	Fentanyl 1 mcg/kg		27/29
Bhardwaj (2007) India [50]	Journal of Postgraduate Medicine	I-II, 1-12 years, GA, Landmark	Hypospadias, urethroplasty	0.25% Bupivacaine 0.75 ml/kg	Control (no adjuvant)	Neostigmine 2 mcg/kg			27/29
Bonisson (2019) Brazil [51]	Brazilian Journal of Anesthesiology	I-II, 1-10 years, GA, Landmark	Hypospadias	0.165% Bupivacaine ml/kg	Control (no adjuvant)	Clonidine 1 mcg/kg			20/20
Chertin (2016) Israel [52]	Current Urology	I-II, 2 months - 14 years, GA, Landmark	Other urological procedures	0.2% Bupivacaine 1.2 ml/kg	Fentanyl 2 mcg/kg	Morphine 15-20 mcg/kg			20/20
Cho (2015) Republic of Korea [53]	Biological & Pharmaceutical Bulletin	I, 1-6 years, GA, Landmark	Orchidopexy	0.15% Ropivacaine 1.5 ml/kg	Control (no adjuvant)	Dexmedetomidine 1 mcg/kg			40/40
Choudhuri (2008) India [54]	Anaesth Intensive Care	I-II, 3-9 years, GA, Landmark	Inguinal hernia	0.25% Bupivacaine 0.5 ml/kg	Control (no adjuvant)	Ketamine 0.5 mg/kg	Tramadol 1 mg/kg		25/25/25
Choudhary (2016) India [55]	Indian Journal of Anesthesia	I-II, 1-5 years, Sedation, Landmark	Inguinal hernia	0.2% Ropivacaine 1 ml/kg	Control (no adjuvant)	Dexamethasone 0.1 mg/kg			64/64
Cook (1995) Scotland [56]	British Journal of Anaesthesia	Not specified, 1-10 years, GA, Landmark	Orchidopexy	0.25% Bupivacaine 1 ml/kg	Clonidine 2 mcg/kg	Ketamine 0.5 mg/kg			20/20
Dogra (2018) India [57]	Indian Journal of Anesthesia	I-II, 2-7 years, GA, Landmark	Inguinal hernia	0.125% Levobupivacaine 1 ml/kg	Control (no adjuvant)	Tramadol 1.5 mcg/kg			26/26
El-Feky^# ^(2015) Egypt [58]	Egyptian Journal of Anesthesia	I-II, 3 to 10 years, GA, Landmark	Inguinal hernia, orchidopexy, hypospadias	0.25% Bupivacaine 0.5 ml/kg	Control (no adjuvant)	Fentanyl 1 mcg/kg	Dexmedetomidine 1 mcg/kg	Dexamethasone 0.1 mg/kg	29/28/28
El-Hennawy (2009) Egypt [59]	British Journal of Anaesthesia	I-II, 6 months - 6 years, GA, Landmark	Other urological procedures, other abdominal procedures	0.25% Bupivacaine 1 ml/kg	Control (no adjuvant)	Dexmedetomidine 2 mcg/kg	Clonidine 2 mcg/kg		20/20/20
El-Shamaa (2016) Egypt [60]	Saudi Journal of Anesthesia	I-II, 1-5 years, GA, Landmark	Inguinal hernia, orchidopexy, hypospadias, urethroplasty	0.25% Bupivacaine 1 ml/kg	Dexmedetomidine 2 mcg/kg	Morphine 30 mcg/kg			25/25
Farrag (2014) Egypt [61]	Urology Annals	I-II, 3-10 years, GA, Landmark	Inguinal hernia, orchidopexy, hypospadias	0.25% Bupivacaine 0.5 ml/kg	Ketamine 0.5 mg/kg	Magnesium 50 mg			20/20
Fernandes^$^ (2012) Brazil [62]	Journal of Anesthesia	I-II, 1-10 years, GA, Landmark	Inguinal hernia, other urological procedures, other abdominal procedures	0.166% Bupivacaine 1.0 ml/kg	Control (no adjuvant)	Morphine 20 mcg/kg	Clonidine 1 mcg/kg		20/20/20
Gaitini (2000) Israel [63]	Anesthesia & Analgesia	I, 1-8 years, GA, Landmark	Inguinal hernia	0.25% Bupivacaine 1.0 ml/kg	Control (no adjuvant)	Fentanyl 1 mcg/kg			30/30
George (2018) India [64]	Journal of Clinical & Diagnostic Research	I-II, 2-6 years, GA, Landmark	Inguinal hernia	0.25% Bupivacaine 1 ml/kg	Control (no adjuvant)	Neostigmine 2 mcg/kg			20/20
Goyal (2016) India [65]	Anesthesia: Essays & Research	I-II, 2-10, GA, Landmark	Inguinal hernia, orchidopexy, hypospadias	0.25% Bupivacaine 1.0 ml/kg	Control (no adjuvant)	Dexmedetomidine 1 mcg/kg			50/50
Gulec (1998) Turkey [66]	European Journal of Anesthesiology	I-II, 1-12 years, GA, Landmark	Inguinal hernia, orchidopexy, circumcision, hydrocele, hypospadias	0.125% Bupivacaine 0.75 ml/kg	Control (no adjuvant)	Morphine 0.05 mg/kg			20/20
Gunes (2004) Turkey [67]	Pediatric Anesthesia	I-II, 1-10 years, GA, Landmark	Inguinal hernia	0.2% Ropivacaine 0.5 ml/kg	Ketamine 1 mcg/kg	Tramadol 1 mg/kg			33/34
Gupta (2003) India [68]	Journal of Anesthesiology Clinical Pharmacology	I, 1-12 years, GA, Landmark	Not stated	0.25% Bupivacaine 1 ml/kg	Control (no adjuvant)	Tramadol 1 mg/kg			20/20
Gupta (2009) India [69]	Journal of Anesthesiology Clinical Pharmacology	I-II, 2-8 years, GA, Landmark	Other urological procedures, other abdominal procedures, orthopedic	0.25% Bupivacaine 0.75 ml/kg	Control (no adjuvant)	Morphine 0.03 mg/kg			25/25
Gupta (2017) India [70]	Indian Journal of Anesthesia	I-II, 1-8 years, GA, Landmark	Inguinal hernia, orchidopexy, hypospadias, other urological procedures	0.25% Ropivacaine 1 ml/kg	Tramadol 2 mg/kg	Dexmedetomidine 2 mcg/kg			30/30
Hegazy (2013) Egypt [71]	Chinese German Journal of clinical Oncology	I-III, 0-5 years, GA, Landmark	Other abdominal procedures	0.1875% Bupivacaine 1 ml/kg	Control (no adjuvant)	Tramadol 1 mg/kg			20/20
Jain (2018) India [72]	Anesthesia, Pain & Intensive Care	I-II, 6 m - 6y, GA, Landmark	Herniotomy, orchidopexy, urethroplasty, others	0.25% Ropivacaine 1 ml/kg	Control (no adjuvant)	Dexmedetomidine 1 mcg/kg			30/30
Joshi (2004) USA [73]	Pediatric Anesthesia	Not specified, 6months-6years, GA, Landmark	Inguinal hernia, orchidopexy, hydrocele	0.125% Bupivacaine 1 ml/kg	Control (no adjuvant)	Clonidine 2mcg/kg			18/18
Kalsotra (2019) India [74]	JK Science	I-II, 1-8 years, GA, Landmark	Other sub-umbilical surgeries	0.2% Ropivacaine 1 ml/kg	Control (no adjuvant)	Dexmedetomidine 2 mcg/kg			30/30
Kamal (2016) India [75]	Saudi Journal of Anesthesia	I-II, 2-10 years, GA, Landmark	Inguinal hernia, orchidopexy, circumcision, urethroplasty, orchidectomy	0.25% Ropivacaine 1 ml/kg	Control (no adjuvant)	Dexmedetomidine 2 mcg/kg			30/30
Karaaslan (2009) Turkey [76]	Pediatric Anesthesia	I, 5months-5years, GA, Landmark	Inguinal hernia, orchidopexy, hypospadias	0.25% Levobupivacaine 1 ml/kg	Control (no adjuvant)	Neostigmine 2 mcg/kg			20/20
Kaur (2016) India [77]	Anesthesia: Essays & Research	I-II, 1-10 years, GA, Landmark	Inguinal hernia, orchidopexy, urethroplasty	0.25% Bupivacaine 1 ml/kg	Control (no adjuvant)	Ketamine 0.5 mg/kg			30/30
Khakurel (2018) Nepal [78]	J Nepal Health Research Council	I-II, 2-7 years, GA, Landmark	Inguinal hernia	0.5% Bupivacaine 1 ml/kg	Control (no adjuvant)	Clonidine 2 mcg/kg			30/30
Khatavkar (2016) India [79]	Anesthesia, Pain & Intensive Care	I-II, 2-10 years, GA, Landmark	Inguinal hernia, orchidopexy, circumcision, urethroplasty, orthopedic	0.25% Ropivacaine 1 ml/kg	Fentanyl 1 mcg/kg	Clonidine 1 mcg/kg			30/30
Kim (2014) South Korea [80]	Pediatric Anesthesia	I-II, 2-6 years, GA, Landmark	Inguinal hernia	0.15% Ropivacaine 1 ml/kg	Control (no adjuvant)	Magnesium 50 mg			37/38
Kim (2014) South Korea [81]	British Journal of Anaesthesia	I-II, 0.5-5 years, GA, Ultrasound	Orchidopexy	0.15% Ropivacaine 1.5 ml/kg	Control (no adjuvant)	Dexamethasone 0.1 mg/kg			38/39
Koul (2009) India [82]	Indian Journal of Anesthesia	I, 1-10 years, GA, Landmark	Inguinal hernia	0.25% Bupivacaine 0.75 ml/kg	Control (no adjuvant)	Clonidine 2 mcg/kg			20/20
Kumar (2005) India [83]	Anesthesia & Analgesia	I, 5-10 years, GA, Landmark	Inguinal hernia	0.25% Bupivacaine 1 ml/kg	Control (no adjuvant)	Ketamine 0.5 mg/kg	Neostigmine 2 mcg/kg		20/20/20
Laha (2012) India [84]	Saudi Journal of Anesthesia	I, 2-11 years, GA, Landmark	Other urological procedures, other abdominal procedures	0.2% Ropivacaine 1 ml/kg	Control (no adjuvant)	Clonidine 2 mcg/kg			15/15
Martindale (2004) UK [85]	British Journal of Anaesthesia	Not specified, 3 months - 6 years, GA, Landmark	Inguinal hernia, orchidopexy	0.25% Bupivacaine 1 ml/kg	Control (no adjuvant)	Ketamine 0.5 mg/kg			20/19
Meenakshi Karuppiah (2016) India [86]	Indian Journal of Anesthesia	I-II, 0.5-8 years, GA, Landmark	Not stated	0.25% Bupivacaine 1 ml/kg	Control (no adjuvant)	Dexmedetomidine 1 mcg/kg			28/28
Memis (2003) Turkey [87]	Paediatric Anesthesia	I, 1-5 years, GA, Landmark	Inguinal hernia, hypospadias	0.25% Bupivacaine 0.5 ml/kg	Control (no adjuvant)	Neostigmine 1 mcg/kg			20/20
Nafiu (2006) Ghana [88]	Journal of the National Medical Association	I-II, 2-8 years, GA, Landmark	Not stated	0.125% Bupivacaine 1 ml/kg	Control (no adjuvant)	Ketamine 0.5 mg/kg			20/20
Narasimhamurthy (2016) India [89]	Journal of Clinical & Diagnostic Research	I, 2-10 years, GA, Landmark	Inguinal hernia, orchidopexy, circumcision	0.2% Ropivacaine 1 ml/kg	Control (no adjuvant)	Clonidine 1 mcg/kg			30/30
Neogi (2010) India [90]	Journal of Anesthesiology Clinical Pharmacology	I, 1-6 years, GA, Landmark	Inguinal hernia	0.25% Ropivacaine 1 mL/kg	Control (no adjuvant)	Clonidine 1 mcg/kg	Dexmedetomidine 1 mcg/kg		25/25/25
Nisa (2019) Pakistan [91]	Anesthesia, Pain & Intensive Care	I-II, 5-10 years, GA, Landmark	Other sub-umbilical surgeries	0.25% Bupivacaine 0.5-1 mL/kg	Control (no adjuvant)	Tramadol 1 mcg/kg			50/54
Odes (2010) Turkey [92]	Agri Dergisi	I-II, 1-4 years, GA, Landmark	Inguinal hernia	0.2% Ropivacaine 1 mL/kg	Control (no adjuvant)	Ketamine 0.5 mg/kg			15/15
Pan (2005) India [93]	Journal of Anesthesiology Clinical Pharmacology	I, 5-10 years, GA, Landmark	Inguinal hernia	0.25% Bupivacaine 1 mL/kg	Control (no adjuvant)	Ketamine 0.5 mg/kg			25/25
Parameswari (2010) India [94]	Indian Journal of Anesthesia	I-II, 1-3 years, GA, Landmark	Inguinal hernia, orchidopexy, circumcision	0.25% Bupivacaine 1 mL/kg	Control (no adjuvant)	Clonidine 1 mcg/kg			50/50
Parameswari (2017) India [95]	Journal of Anesthesiology Clinical Pharmacology	I-II, 0.5-6 years, GA, Landmark	Inguinal hernia, orchidopexy, circumcision, hypospadias, other urological procedures, other abdominal procedures, orthopedic	0.125% Bupivacaine 1 mL/kg	Control (no adjuvant)	Dexamethasone 0.1 mg/kg			65/65
Pathania (2003) India [96]	Journal of Anesthesiology Clinical Pharmacology	I, 3-12 years, GA, Landmark	Not stated	0.25% Bupivacaine 1 mL/kg	Control (no adjuvant)	Ketamine 0.5 mg/kg			20/20
Paul (2010) India [97]	Pharmacology Online	I, 1-6 years, GA, Landmark	Inguinal hernia	0.25% Bupivacaine 1 mL/kg	Control (no adjuvant)	Clonidine 1 mcg/kg	Neostigmine 2 mcg/kg		25/25/25
Potti (2017) India [98]	Anesthesia: Essays & Research	I-II, 2-12 years, GA, Landmark	Inguinal hernia, hypospadias, other urological procedures, other abdominal procedures	0.25% Levobupivacaine 1 mL/kg	Control (no adjuvant)	Clonidine 1 mcg/kg			25/25
Prakash (2006) India [99]	British Journal of Anaesthesia	I-II, 2 to 8 years, GA, Landmark	Inguinal hernia	0.25% Bupivacaine 0.75 mL/kg	Control (no adjuvant)	Tramadol 1 mg/kg	Tramadol 1.5 mg/kg	Tramadol 2 mg/kg	20/20
Priolkar (2016) India [100]	JCDR	I, 1 to 10 years, GA, Landmark	Inguinal hernia, orchidopexy, circumcision, hypospadias	0.125% Bupivacaine 1 mL/kg	Control (no adjuvant)	Clonidine 1 mcg/kg			30/30
Rawat (2019) India [101]	Anesthesia Essays & Research	I-II, 1-10 years, GA, Landmark	Perineal surgery	0.25% Levobupivacaine 1 mL/kg	Control (no adjuvant)	Tramadol 1 ml/kg	Clonidine 1 mcg/kg		22/22/22
Ribeiro Jr (2011) Brazil [102]	African Journal of Pharmacy & Pharmacology	I-II, 2 to 8 years, Sedation,	Inguinal hernia, orchidopexy, circumcision	0.25% Bupivacaine 0.75 mL/kg	Control (no adjuvant)	Clonidine 1 mcg/kg	Ketamine 0.5 mg/kg		10/21/20
Saadawy (2009) Egypt [103]	Acta Anaesthesiologica Scandinavica	I, 1-6 years, GA, Landmark	Inguinal hernia, orchidopexy	0.25% Bupivacaine 1 mL/kg	Control (no adjuvant)	Dexmedetomidine 1 mcg/kg			30/30
Sanwatsarkar (2017) India [104]	Journal of Anesthesiology & Clinical Pharmacology	I-II, 1 to 7 years, GA, Landmark	Inguinal hernia, orchidopexy, circumcision, urethroplasty, other urological procedures, other abdominal procedures	0.25% Bupivacaine 1 mL/kg	Control (no adjuvant)	Clonidine 1 mcg/kg			25/25
Sarvesh (2019) India [105]	Journal of Clinical & Diagnostic Research	I-II, 2-12 years, GA, USG	Other infra-inguinal procedures	0.25% Ropivacaine 1 mL/kg	Control (no adjuvant)	Dexmedetomidine 1 mcg/kg			30/30
Sayed (2018) Egypt [106]	Korean Journal of Pain	I-II, 3-10 years, GA, Landmark	Other lower abdominal procedures, ectopic kidney, kidney stone, cystolithotomy, re-implantation of ureter	0.25% Bupivacaine 1 mL/kg	Control (no adjuvant)	Tramadol 1 mcg/kg			30/30
Sayed (2018) Egypt [107]	Egyptian Journal of Anesthesia	I-II, Not stated, GA, Landmark	Inguinal hernia, orchidopexy, hypospadias, other urological surgery, other infra-inguinal procedures	0.25% Bupivacaine 1 mL/kg	Control (no adjuvant)	Dexmedetomidine 1 mcg/kg			30/30
Senel (2001) Turkey [108]	Acta Anaesthesiologica Scandinavica	I, 1-7 years, GA, Landmark	Inguinal hernia	0.25% Bupivacaine 1 mL/kg	Control (no adjuvant)	Tramadol 1.5 mg/kg			20/20
Sharpe (2001) UK [109]	Paediatric Anesthesia	I-II, Not mentioned, GA, Landmark	Circumcision	0.25% Bupivacaine 0.5 mL/kg	Control (no adjuvant)	Clonidine 1 mcg/kg			25/24
She (2015) China [110]	Journal of Clinical Anesthesia	I-II, 1 & 3 years, Sedation, Landmark	Inguinal hernia, hydrocele	0.2% Levobupivacaine 1 mL/kg	Control (no adjuvant)	Dexmedetomidine 2			70/70
Shirmohammadie (2019) Iran [111]	Acta Biomed	I-II, 1-3 years, GA, Landmark	Inguinal hernia, hypospadias, urethroplasty	0.25% Bupivacaine 1 mL/kg	Control (no adjuvant)	Neostigmine 2 mcg/kg	Ketamine 0.5 mg/kg		20/20/20
Shrestha (2010) Nepal [112]	Journal of Nepal Health Research Council	I, 1-6 years, GA, Landmark	Inguinal hernia, circumcision	0.25% Bupivacaine 0.5 mL/kg	Control (no adjuvant)	Tramadol 1 mg/kg			20/20
Singh (2010) India [113]	British Journal of Anaesthesia	I-II, 1 to 6 years, GA, Landmark	Other abdominal procedures	0.2% Bupivacaine 1.25 mL/kg	Clonidine 2 mcg/kg	Morphine 30 mcg/kg			30/30/30
Singh (2012) Nepal [114]	Journal of Nepal Paediatric Society	I-II, 1 to 10 years, GA, Landmark	Not stated	0.2% Ropivacaine 0.75 mL/kg	Control (no adjuvant)	Ketamine 0.5 mg/kg	Fentanyl 1 mcg/kg		25/25
Sinha (2016) India [115]	Anesthesia Essays & Research	I-II, 1-6 years, GA, Landmark	Orchidopexy, circumcision, hydrocele, hypospadias, urethroplasty	0.25% Bupivacaine 0.5 mL/kg	Dexamethasone 0.1 mg/ kg	Clonidine 1 mcg/kg			30/30
Solanki (2016) India [116]	Saudi Journal of Anesthesia	I-II, 1-12 years, GA, Landmark	Inguinal hernia, orchidopexy, hypospadias, anorectoplasty	0.25% Bupivacaine 1 mL/kg	Tramadol 2 mg/kg	Fentanyl 2 mcg/kg			50/50
Sridhar (2017) India [117]	Anesthesia Essays & Research	I-II, 3 to 12 years, GA, Landmark	Not stated	0.2% Ropivacaine 0.5 mL/kg	Control (no adjuvant)	Dexmedetomidine 1 mcg/kg	Dexamethasone 0.1 mg/kg	Magnesium sulfate 50 mg	32/32/32/32
Srinivasan (2016) India [118]	Indian Journal of Anesthesia	I-II, 4-10 years, GA, Landmark	Inguinal hernia	0.15% Ropivacaine 1.5 mL/kg	Control (no adjuvant)	Dexamethasone 0.1 mg/kg			35/35
Taheri (2010) Iran [119]	Pediatric Anesthesia	I, 1-7years, GA, Landmark	Inguinal hernia	0.25% Bupivacaine 0.9 mL/kg	Neostigmine 2 mcg/kg	Tramadol 1 mg/kg			30/30
Turan (2003) Turkey [120]	Anesthesiology	I, 1-6 years, GA, Landmark	Inguinal hernia, hypospadias	0.2% Ropivacaine 0.5 mL/kg	Control (no adjuvant)	Neostigmine 2 mcg/kg			22/22
Vakkapatti (2019) India [121]	Open Pain Journal	I-II, 0-3 years, GA, Landmark	Other infra-inguinal procedures	0.25% Levobupivacaine 2 mL/kg	Control (no adjuvant)	Fentanyl 1 mcg/kg			30/30
Vetter^$ ^(2007) USA [122]	Anesthesia & Analgesia	I-II, 6 months to 6 years, GA, Landmark	Ureteric reimplantation	0.2% Ropivacaine 1 mL/kg	Clonidine 2 mcg/kg	Morphine 50 mcg/kg			20/20
Weber (2003) Germany [123]	Pediatric Anesthesia	I-II, 1 month to 9 years, GA, Landmark	Inguinal hernia, orchidopexy, circumcision	0.125% Bupivacaine 1 ml/kg	Control (no adjuvant)	Ketamine 0.5 mg/kg			15/15
Xiang (2013) China [124]	British Journal of Anaesthesia	I, 1 - 6 years, Sedation, Landmark	Inguinal hernia	0.25% Bupivacaine 1 mL/kg	Control (no adjuvant)	Dexmedetomidine 1 mcg/kg			30/30
Yao (2018) China [125]	Pediatric Anesthesia	I, 2-5 years, GA, Landmark	Not stated	0.25% Levobupivacaine 1 ml/kg	Control (no adjuvant)	Dexmedetomidine 1 mcg/kg			30/30
Yildiz (2006) Turkey [126]	Acta Anaesthesiologica Scandinavica	I-II, 1-10, GA, Ultrasound	Inguinal hernia	0.125% Bupivacaine 1 mL/kg	Control (no adjuvant)	Clonidine 1 mcg/kg	Clonidine 1.5 mcg/kg	Clonidine 2 mcg/kg	15/15
Yildiz (2010) Turkey [127]	Pediatric Anesthesia	I-II, 1-7 years, GA, Landmark	Inguinal hernia	0.125% Levobupivacaine 1 mL/kg	Control (no adjuvant)	Tramadol 1.5 mg/kg			23/23
Yousef (2014) Egypt [128]	Anesthesia: Essays & Research	I-II, 1-6 year, GA, Landmark	Inguinal hernia	0.15% Ropivacaine 1.5 mL/kg	Control (no adjuvant)	Magnesium 50 mg	Dexamethasone 0.1 mg/kg		35/35/35

**Table 2:** Analgesic regimen in the included RCTs. ASA Class, American Society of Anesthesiology classification; GA, general anesthesia; IM, intramuscular; IV, intravenous; mcg/kg, microgram per kilogram; mg, milligram; mg/kg, milligram per kilogram; mL/kg, milliliter per kilogram; PO, per oral; supp, suppository; syp, syrup; RCT, randomized control trial Pain scales: CHEOPS, Children of Eastern Ontario Pain Scale; CHIPPS, Children & Infants Postoperative Pain; CRIES, Crying, Oxygenation, Vital Signs, Facial Expression, & Sleeplessness; FLACC, Face, Legs, Activity, Crying & Consolability Scale; FPSR, Facial Pain Scale-Revised; mCHEOPS, Modified CHEOPS; MOPS, Modified Objective Pain Scale; OPDS, Objective Pain Discomfort Score; OPS, Objective Pain Score; OsPS, Observational Pain Score; PDS, Pain Discomfort Score; TPPPS, Modified Toddler Pre-schooler Postoperative Pain Scale; VrPS, Verbal Pain Score; WBFS, Wong-Baker Faces Scale

Name, year, country	Premedication	Intraoperative sedation	Pain scale used	Rescue analgesia	Postoperative analgesia
Abu-Elyazed (2017) Egypt [40]	None	IV Fentanyl 1 mcg/kg; Patients were excluded	MOPS	MOPS ≥ 4	IV Acetaminophen 15 mg/kg
Ahuja (2014) India [41]	Oral Midazolam 0.4 mg/kg	None	Facies scale (if age ≤ 5 years); Modified VAS (if age > 5 years)	VAS ≥ 3	Oral Acetaminophen 15 mg/kg
Ahuja (2015) India [42]	Oral Midazolam 0.5 mg/kg	None	FLACC (if age ≤ 5); Modified VAS (if age > 5)	VAS > 4	Oral or rectal Acetaminophen 20 mg/kg
Akin (2010) Turkey [43]	Oral Midazolam 0.5 mg/kg	None	CHIPPS	CHIPPS ≥ 4	Oral Tramadol 2 mg/kg
Al-Zaben (2015) Jordan [44]	None	IV Fentanyl 1 mcg/kg	MOPS	MOPS ≥ 4	Oral Acetaminophen 15 mg/kg
Al-Zaben (2016) Jordan [45]	None	IV Fentanyl 1 mcg/kg	OPS	OPS ≥ 4	Oral Acetaminophen 15 mg/kg
Aliena (2018) India [46]	IV Midazolam 0.05 mg/kg & IV Fentanyl 2 mcg/kg	Supp Acetaminophen 20mg/kg to all	MOPS	MOPS > 3	OPS > 3, Syp. Ibuprofen 5mg/kg
Amitha (2019) India [47]	Syp Promethazine 1 mg/kg night before	None	OPS	OPS ≥ 6	OPS>=6, Supp Acetaminophen 20 mg/kg
Anand (2011) India [48]	Oral Midazolam 0.5 mg/kg	None	FLACC	FLACC ≥ 4	Syp Acetaminophen 15 mg/kg
Aziz (2016) Egypt [49]	None	IV Fentanyl; dose not defined	Not defined	Not stated	IV Acetaminophen 15 mg/kg
Bhardwaj (2007) India [50]	Oral Midazolam 0.5 mg/kg	None	OPS if age < 5 years; VAS used if age > 5 years	OPS ≥ 4	Oral Acetaminophen 15 mg/kg
Bonisson (2019) Brazil [51]	None	None	FLACC	Patient or guardian request	IV Morphine 20 - 50 mcg/kg
Chertin (2016) Israel [52]	None	None	FLACC if age < 3; WBFS if age ≥ 3	Not stated	Acetaminophen, Ibuprofen & Morphine; Dose Not Stated
Cho (2015) Republic of Korea [53]	None	None	FLACC & CHEOPS	FLACC ≥ 4; CHEOPS ≥ 4	IV Fentanyl 0.5 mcg/kg (PACU); Oral Acetaminophen (ward)
Choudhuri (2008) India [54]	None	Pethidine 1 mg/kg initially & subsequently 0.5 mg/kg	PDS	PDS > 4	Oral Acetaminophen 10 mg/kg
Choudhary (2016) India [55]	Midazolam 0.05 mg/kg & Glycopyrrolate 0.08 mg/kg	Ketamine 2m/kg	FLACC	FLACC ≥ 4.	Supp Acetaminophen 15 mg/kg
Cook (1995) Scotland [56]	None	Not stated	MOPS	OPS > 4	Oral Acetaminophen 10 mg/kg
Dogra (2018) India [57]	IV Midazolam 0.05 mg/kg	None	CHIPPS	CHIPPS > 4	Supp Acetaminophen 30mg/kg
El-Feky^#^ (2015) Egypt [58]	None	None	MOPS	MOPS > 4	Acetaminophen 15 mg/kg
El-Hennawy (2009) Egypt [59]	Oral Midazolam 0.5 mg/kg	IV Fentanyl 1 mcg/kg	FLACC	FLACC ≥ 4	IM Morphine 0.2 mg/kg
El-Shamaa (2016) Egypt [60]	IM Ketamine 1 mg/kg & atropine 0.01 mg/kg	IV Fentanyl 1 mcg/kg	FLACC	FLACC ≥ 4	Not Stated
Farrag (2014) Egypt [61]	None	None	VAS	VAS > 3	VAS>3, Rectal Acetaminophen 15mg/kg, VAS>6, IV Pethidine 1mg/kg
Fernandes^$^, (2012) Brazil [62]	None	None	FLACC	Not stated	Metamizole, Ibuprofen, Morphine
Gaitini (2000) Israel [63]	None	None	mCHEOPS	mCHEOPS score > 5	IV Fentanyl 1 mcg/kg (PACU); 15 mg/kg Acetaminophen (Ward)
George (2018) India [64]	Syp Pedicloryl 75 mg/kg	None	PDS	PDS > 4	Supp Acetaminophen 15 mg/kg
Goyal (2016) India [65]	Glycopyrrolate 0.04 mg/kg & ondansetron 0.1 mg/kg	None	FLACC	FLACC ≥ 7	Supp Acetaminophen 10 mg/kg
Gulec (1998) Turkey [66]	None	None	VrPS	VrPS ≥ 3	Rectal Acetaminophen 50-100 mg/kg
Gunes (2004) Turkey [67]	None	None	CHEOPS	CHEOPS ≥ 7	Oral Acetaminophen 15 mg/kg
Gupta (2003) India [68]	Oral trimethazine 3 mg/kg	None	OPDS	OPS ≥ 6	Oral Acetaminophen 20 mg/kg
Gupta (2009) India [69]	Oral Midazolam 0.2 mg/kg	None	TPPPS	TPPPS > 4	IM Acetaminophen 3-5 mg/kg
Gupta (2017) India [70]	IV Midazolam 0.05 mg/kg	None	FLACC	FLACC ≥ 4	Supp Acetaminophen 15 mg/kg
Hegazy (2013) Egypt [71]	Not stated	Fentanyl 2 mcg/kg, Morphine 0.1 mg/kg	FLACC	Parents’ request or FLACC > 3.	IV Acetaminophen 10 mg/kg & IV Tramadol 1 mg/kg q8h
Jain (2018) India [72]	IV Midazolam 0.05 mg/kg	None	FLACC	FLACC ≥ 4	Syrup Acetaminophen 15 mg/kg
Joshi (2004) USA [73]	None	Not stated	Faces scale in PACU, VAS at home	Moderate to severe pain	IV Fentanyl 5-10mcg PRN
Kalsotra (2019) India [74]	None	None	ObPS	ObPS > 4	Supp Acetaminophen 20 mg/kg or IV Diclofenac 1mg/kg
Kamal (2016) India [75]	oral Midazolam 0.5 mg/kg	None	FLACC	FLACC ≥ 4	Oral Acetaminophen 10 mg/kg
Karaaslan (2009) Turkey [76]	Oral Midazolam 0.5m/kg	Not stated	CHIPPS	CHIPPS >10	Rectal Acetaminophen 20mg/kg
Kaur (2016) India [77]	None	None	OPS	OPS ≥ 4	Oral Acetaminophen 15 mg/kg
Khakurel (2018) Nepal [78]	None	None	FLACC	FLACC ≥ 4	IV Acetaminophen 15 mg/kg
Khatavkar (2016) India [79]	Oral Midazolam 0.5 mg/kg; IV pentazocine 0.3 mg/kg	None	FLACC	FLACC > 4	IV Acetaminophen 15 mg/kg
Kim (2014) South Korea [80]	None	None	FLACC	FLACC ≥ 5	Fentanyl 0.5 mcg/kg
Kim (2014) South Korea [81]	None	1mcg/kg Fentanyl, excluded	CHEOPS & FLACC	CHEOPS & FLACC > 4 (PACU); NRS > 4 (home)	IV 0.5 mcg/kg Fentanyl (PACU); Oral Ibuprofen 5 mg/kg (home)
Koul (2009) India [82]	None	None	OPS	OPS > 4	Oral Acetaminophen 10 mg/kg
Kumar (2005) India [83]	None	Fentanyl 2 mcg/kg	VrPS	VrPS > 4	Oral Acetaminophen 20 mg/kg
Laha (2012) India [84]	Nasal Midazolam 0.2 mg/kg	Not stated	CHEOPS	CHEOPS > 4	IM Pethidine 1 mg/kg
Martindale (2004) UK [85]	paracetamol 20 mg/kg; local tetracaine	Rectal diclofenac 1 mg/kg	MOPS	OPS ≥4	Oral Acetaminophen 15 mg/kg
Meenakshi Karuppiah (2016) India [86]	oral triclofos 100 mg/kg; oral atropine 0.03 mg/kg	None	FLACC	FLACC ≥ 4	Rectal Diclofenac 1–2 mg/kg; Oral Ibuprofen 4–8 mg/kg
Memis (2003) Turkey [87]	rectal Midazolam 0.4 mg/kg	None	TPPPS	TPPPS > 3	Rectal Acetaminophen 20 mg/kg
Nafiu (2006) Ghana [88]	None	None	Hannallah Observational Pain Score	Score > 4	IV Morphine 0.1 mg/kg (PACU); Acetaminophen 15 mg/kg (ward)
Narasimhamurthy (2016) India [89]	Oral Midazolam 0.5 mg/kg	None	FLACC	FLACC > 4	Oral Acetaminophen 15 mg/kg
Neogi (2010) India [90]	Oral Midazolam 0.5 mg/kg	None	CRIES	CRIES ≥ 4	Oral Acetaminophen
Nisa (2019) Pakistan [91]	Not stated	Not stated	FLACC	Not stated	Not Stated
Odes (2010) Turkey [92]	None	None	mCHEOPS	CHEOPS ≥ 4	Rectal Acetaminophen 20 mg/kg
Pan (2005) India [93]	None	None	VrPS	VrPS > 4	Acetaminophen 20 mg/kg
Parameswari (2010) India [94]	Oral Midazolam 0.5 mg/kg	IV Fentanyl 1 mcg/kg	FLACC	FLACC ≥ 4	Rectal Acetaminophen 40 mg/kg Loading Dose, then 20 mg/kg q6h
Parameswari (2017) India [95]	Oral Midazolam 0.5 mg/kg	IV Fentanyl 2 mcg/kg	FLACC	FLACC > 3	Oral Acetaminophen 15 mg/kg
Pathania (2003) India [96]	Oral promethazine 0.5 mg/kg	None	ObPS	ObPS > 6	Acetaminophen 15 mg/kg
Paul (2010) India [97]	Oral Midazolam 0.5 mg/kg	None	CRIES	CRIES ≥ 4	Oral Acetaminophen
Potti (2017) India [98]	Oral promethazine 1 mg/kg	IV Fentanyl 2 mcg/kg	CHIPPS	CHIPPS ≥ 4	IV Acetaminophen 10 mg/kg
Prakash (2006) India [99]	None	None	PDS	PDS > 4	Oral Acetaminophen 10 mg/kg
Priolkar (2016) India [100]	Oral Midazolam 0.75 mg/kg	None	VrPS	VrPS ≥ 4	Syp Acetaminophen 15mg/kg
Rawat (2019) India [101]	IV Midazolam 0.05 mg/kg	None	CHIPPS	CHIPPS > 4	Not Stated
Ribeiro Jr (2011) Brasil [102]	None	Not stated	Oucher Pain Scale	Not stated	Dipyrone 30 mg/kg
Saadawy (2009) Egypt [103]	None	None	OPS	OPS > 4	Oral Acetaminophen 10 mg/kg
Sanwatsarkar (2017) India [104]	Oral Midazolam 0.5 mg/kg	Fentanyl 2 mcg/kg	FLACC	FLACC ≥ 4	Supp Acetaminophen 40 mg/kg
Sarvesh (2019) India [105]	Not stated	None	FLACC	FLACC ≥ 4	Syrup Acetaminophen 10 mg/kg
Sayed (2018) Egypt [106]	Oral Midazolam 0.05 mg/kg	Not stated	FLACC	FLACC > 4	Acetaminophen 15 mg/kg
Sayed (2018) Egypt [107]	Not stated	Not stated	FLACC	FLACC ≥ 3	IV Acetaminophen 15 mg/kg
Senel (2001) Turkey [108]	None	None	OPS	Not stated	Suppository Acetaminophen 10 mg/kg
Sharpe (2001) UK	None	Not stated	ObPS	Not stated	Oral Acetaminophen 15mg/kg
She (2015) China [110]	None	Midazolam 0.1 mg/kg & Propofol 4mg/kg/hr	CHIPPS	CHIPPS > 4	Oral Ibuprofen 10 mg/kg
Shirmohammadie (2019) Iran [111]	None	None	FPSR	FPSR ≥ 4	Supp Acetaminophen 125 mg q6h for 24h; Rescue with IV Meperidine 0.3 mg/kg
Shrestha (2010) Nepal [112]	None	None	Modification of pain/discomfort scale	Not stated	Not Stated
Singh (2010) India [113]	None	Fentanyl 2 mcg/kg	FLACC	FLACC ≥ 4	IV Fentanyl 1 mcg/kg & Supp Acetaminophen 40 mg/kg
Singh (2012) Nepal [114]	Oral atropine 0.02 mg/kg	Midazolam 0.1 mg/kg	FLACC	FLACC ≥ 4	Oral Acetaminophen 10 mg/kg
Sinha (2016) India [115]	Oral Pedicloryl (Triclofos) 100 mg/kg	Fentanyl 1 mcg/kg	FLACC	FLACC > 4	Oral Acetaminophen 15 mg/kg
Solanki (2016) India [116]	None	None	FLACC	FLACC > 4	Not Stated
Sridhar (2017) India [117]	Not stated	IV Fentanyl 1 mcg/kg; Patients were excluded	MOPS	MOPS > 4	IV Acetaminophen 15 mg/kg
Srinivasan (2016) India [118]	IV atropine 0.01mg/kg	IV Midazolam 0.05mg/kg, IV Fentanyl 1.5mc/kg	VAS	VAS > 4	IV Acetaminophen 15mg/kg
Taheri (2010) Iran [119]	None	Fentanyl 2mcg/kg	FLACC	FLACC > 4	Rectal Acetaminophen 20-40 mg/kg
Turan (2003) Turkey [120]	None	Alfentanil 10 mcg/kg (block failure)	TPPPS	TPPPS > 3	Rectal Acetaminophen 20 mg/kg
Vakkapatti (2019) India [121]	Oral Midazolam 0.02 mg/kg	IV Tramadol 1 mg/kg or Supp Acetaminophen 20 mg/kg; Patients were excluded	CHIPPS	CHIPPS > 4	IV Tramadol 1 mg/kg or Supp Acetaminophen 20 mg/kg
Vetter^$ ^ (2007) USA [122]	Oral Midazolam 0.5 mg/kg	None	FLACC	FLACC ≥ 4	IV Morphine 30 mcg/kg
Weber (2003) Germany [123]	Rectal Midazolam 0.3 mg/kg	None	ObPS	ObPS > 3	Rectal Acetaminophen 20 mg/kg
Xiang (2013) China [124]	Oral Midazolam 0.5 mg/kg	Ketamine 2 mg/kg	CHIPPS	CHIPPS > 3	IV Fentanyl 0.5 mcg/kg
Yao (2018) China [125]	Oral Midazolam 0.05 mg/kg	None	CHIPPS	CHIPPS ≥ 4	IV Morphine 25 mcg/kg
Yildiz (2006) Turkey [126]	Rectal Midazolam 0.5mg/kg	None	mCHEOPS <5 yr, VAS >5 yr	mCHEOPS > 5, VAS > 30 mm	Rectal Acetaminophen 15 mg/kg
Yildiz (2010) Turkey [127]	Oral Midazolam 0.5 mg/kg	None	CHIPPS	CHIPPS ≥ 4	Rectal Acetaminophen 30 mg/kg
Yousef (2014) Egypt [128]	None	None	CHEOPS & FLACC	CHEOPS & FLACC ≥ 4	IM Pethidine 1 mg/kg

**Table 3:** Outcome characteristics of included studies. DoA, duration of analgesia; NoA, number of doses; ToA, total analgesic requirement Pain scales: CHEOPS, Children of Eastern Ontario Pain Scale; CHIPPS, Children & Infants Postoperative Pain; CRIES, Crying, Oxygenation, Vital Signs, Facial Expression, & Sleeplessness; FLACC, Face, Legs, Activity, Crying & Consolability Scale; FPSR, Facial Pain Scale-Revised; mCHEOPS, Modified CHEOPS; MOPS, Modified Objective Pain Scale; OPDS, Objective Pain Discomfort Score; OPS, Objective Pain Score; OsPS, Observational Pain Score; PDS, Pain Discomfort Score; TPPPS, Modified Toddler Pre-schooler Postoperative Pain Scale; VrPS, Verbal Pain Score; WBFS, Wong-Baker Faces Scale

Name, year, country	Rescue analgesia	Definition of duration of analgesia	DoA	NoA	ToA
Abu-Elyazed (2017) Egypt [40]	MOPS ≥ 4	Time from caudal block to post-operative rescue analgesia.	Yes	No	Yes
Ahuja (2014) India [41]	VAS ≥ 3	Not defined	Yes	No	No
Ahuja (2015) India [42]	VAS > 4	Not defined	Yes	No	No
Akin (2010) Turkey [43]	CHIPPS ≥ 4	Time from caudal block to post-operative rescue analgesia.	Yes	No	No
Al-Zaben (2015) Jordan [44]	MOPS ≥ 4	Time from caudal block to post-operative rescue analgesia.	Yes	No	No
Al-Zaben (2016) Jordan [45]	OPS ≥ 4	Time from caudal block to post-operative rescue analgesia.	Yes	Yes	No
Aliena (2018) India [46]	MOPS > 3	Time from caudal block to post-operative rescue analgesia.	Yes	No	No
Amitha (2019) India [47]	OPS ≥ 6	Time from caudal block to post-operative rescue analgesia.	Yes	Yes	No
Anand (2011) India [48]	FLACC ≥ 4	Time from caudal block to post-operative rescue analgesia.	Yes	No	No
Aziz (2016) Egypt [49]	Not stated	Not defined	Yes	No	Yes
Bhardwaj (2007) India [50]	OPS ≥ 4	Time from caudal block to post-operative rescue analgesia.	Yes	Yes	No
Bonisson (2019) Brazil [51]	Patient or guardian request	Time from caudal block to post-operative rescue analgesia.	Yes	No	Yes
Chertin (2016) Israel [52]	Not stated	Time from caudal block to post-operative rescue analgesia.	Yes	No	Yes
Cho (2015) Republic of Korea [53]	FLACC ≥ 4; CHEOPS ≥ 4	Not defined	Yes	No	No
Choudhuri (2008) India [54]	PDS > 4	Time from caudal block to PDS > 2.	Yes	Yes	Yes
Choudhary (2016) India [55]	FLACC ≥ 4.	Time from caudal block to post-operative rescue analgesia.	Yes	No	No
Cook (1995) Scotland [56]	OPS > 4	Not defined	Yes	Yes	No
Dogra (2018) India [57]	CHIPPS > 4	Time from caudal block to post-operative rescue analgesia.	Yes	Yes	No
El-Feky^#^ (2015) Egypt [58]	MOPS > 4	Time from caudal block to post-operative rescue analgesia.	Yes	No	No
El-Hennawy (2009) Egypt [59]	FLACC ≥ 4	Time from caudal block to post-operative rescue analgesia.	Yes	No	No
El-Shamaa (2016) Egypt [60]	FLACC ≥ 4	Time from caudal block to post-operative rescue analgesia.	Yes	No	No
Farrag (2014) Egypt [61]	VAS > 3	Time from caudal block to post-operative rescue analgesia.	Yes	No	No
Fernandes^$ ^(2012) Brazil [62]	Not stated	Time from caudal block to post-operative rescue analgesia.	Yes	No	No
Gaitini (2000) Israel [63]	mCHEOPS score > 5	Not defined	Yes	No	No
George (2018) India [64]	PDS > 4	Time from caudal block to post-operative rescue analgesia.	Yes	Yes	No
Goyal (2016) India [65]	FLACC ≥ 7	Time from caudal block to post-operative rescue analgesia.	Yes	Yes	Yes
Gulec (1998) Turkey [66]	VrPS ≥ 3	Time from caudal block to pain or post-operative rescue analgesia.	Yes	No	No
Gunes (2004) Turkey [67]	CHEOPS ≥ 7	Time from caudal block to post-operative rescue analgesia.	Yes	No	No
Gupta (2003) India [68]	OPS ≥ 6	Time from caudal block to post-operative rescue analgesia.	Yes	No	No
Gupta (2009) India [69]	TPPPS > 4	Not defined	Yes	No	No
Gupta (2017) India [70]	FLACC ≥ 4	Time from caudal block to post-operative rescue analgesia.	Yes	No	No
Hegazy (2013) Egypt [71]	Parents’ request or FLACC > 3.	Time from caudal block to post-operative rescue analgesia.	Yes	No	No
Jain (2018) India [72]	FLACC ≥ 4	Time from caudal block to post-operative rescue analgesia.	Yes	Yes	No
Joshi (2004) USA [73]	Moderate to severe pain	Time from caudal block to post-operative rescue analgesia.	Yes	Yes	No
Kalsotra (2019) India [74]	ObPS > 4	Time from caudal block to post-operative rescue analgesia.	Yes	Yes	No
Kamal (2016) India [75]	FLACC ≥ 4	Time from caudal block to post-operative rescue analgesia.	Yes	Yes	No
Karaaslan (2009) Turkey [76]	CHIPPS >10	Time from caudal block to post-operative rescue analgesia.	Yes	No	Yes
Kaur (2016) India [77]	OPS ≥ 4	Time from caudal block to post-operative rescue analgesia.	Yes	No	No
Khakurel (2018) Nepal [78]	FLACC ≥ 4	Time from caudal block to post-operative rescue analgesia.	Yes	No	No
Khatavkar (2016) India [79]	FLACC > 4	Time from caudal block to PDS > 2.	Yes	No	No
Kim (2014) South Korea [80]	FLACC ≥ 5	Not defined	Yes	Yes	No
Kim (2014) South Korea [81]	CHEOPS & FLACC > 4 (PACU); NRS > 4 (home)	Not defined	No	Yes	No
Koul (2009) India [82]	OPS > 4	Time from caudal block to first pain post-operatively.	Yes	No	No
Kumar (2005) India [83]	VrPS > 4	Time from caudal block to VrPS > 2.	Yes	No	No
Laha (2012) India [84]	CHEOPS > 4	Not defined	Yes	No	No
Martindale (2004) UK [85]	OPS ≥4	Time from caudal block to post-operative rescue analgesia.	Yes	Yes	Yes
Meenakshi Karuppiah (2016) India [86]	FLACC ≥ 4	Time from caudal block to post-operative rescue analgesia.	Yes	No	No
Memis (2003) Turkey [87]	TPPPS > 3	Time from caudal block to post-operative rescue analgesia.	Yes	No	No
Nafiu (2006) Ghana [88]	Score > 4	Time from caudal block to post-operative rescue analgesia.	Yes	No	No
Narasimhamurthy (2016) India [89]	FLACC > 4	Time from caudal block to post-operative rescue analgesia.	Yes	Yes	Yes
Neogi (2010) India [90]	CRIES ≥ 4	Not defined	Yes	No	No
Nisa (2019) Pakistan [91]	Not stated	Not defined	Yes	No	No
Odes (2010) Turkey [92]	CHEOPS ≥ 4	Not defined	Yes	No	No
Pan (2005) India [93]	VrPS > 4	Time from caudal block to post-operative rescue analgesia.	Yes	No	No
Parameswari (2010) India [94]	FLACC ≥ 4	Time from caudal block to post-operative rescue analgesia.	Yes	Yes	No
Parameswari (2017) India [95]	FLACC > 3	Time from caudal block to post-operative rescue analgesia.	Yes	Yes	Yes
Pathania (2003) India [96]	ObPS > 6	Not defined	Yes	No	No
Paul (2010) India [97]	CRIES ≥ 4	Not defined	Yes	No	No
Potti (2017) India [98]	CHIPPS ≥ 4	Time from caudal block to post-operative rescue analgesia.	Yes	No	No
Prakash (2006) India [99]	PDS > 4	Time from caudal block to post-operative rescue analgesia.	Yes	Yes	Yes
Priolkar (2016) India [100]	VrPS ≥ 4	Time from caudal block to VrPS > 2.	Yes	Yes	No
Rawat (2019) India [101]	CHIPPS > 4	Not defined	Yes	No	No
Ribeiro Jr (2011) Brazil [102]	Not stated	Not defined	Yes	No	No
Saadawy (2009) Egypt [103]	OPS > 4	Time from caudal block to post-operative rescue analgesia.	Yes	Yes	No
Sanwatsarkar (2017) India [104]	FLACC ≥ 4	Time from caudal block to post-operative rescue analgesia.	Yes	Yes	No
Sarvesh (2019) India [105]	FLACC ≥ 4	Time from caudal block to post-operative rescue analgesia.	Yes	Yes	No
Sayed (2018) Egypt [106]	FLACC > 4	Time from caudal block to post-operative rescue analgesia.	Yes	No	Yes
Sayed (2018) Egypt [107]	FLACC ≥ 3	Time from caudal block to post-operative rescue analgesia.	Yes	Yes	Yes
Senel (2001) Turkey [108]	Not stated	Time from caudal block to post-operative rescue analgesia.	Yes	Yes	No
Sharpe (2001) UK [109]	Not stated	Time from caudal block to post-operative rescue analgesia.	Yes	No	No
She (2015) China [110]	CHIPPS > 4	Time from caudal block to post-operative rescue analgesia.	Yes	No	No
Shirmohammadie (2019) Iran [111]	FPSR ≥ 4	Time from caudal block to post-operative rescue analgesia.	Yes	No	Yes
Shrestha (2010) Nepal [112]	Not stated	Time from caudal block to post-operative rescue analgesia.	Yes	Yes	No
Singh (2010) India [113]	FLACC ≥ 4	Time from caudal block to post-operative rescue analgesia.	Yes	No	No
Singh (2012) Nepal [114]	FLACC ≥ 4	Time from caudal block to post-operative rescue analgesia.	Yes	No	No
Sinha (2016) India [115]	FLACC > 4	Not defined	Yes	Yes	No
Solanki (2016) India [116]	FLACC > 4	Not defined	Yes	No	No
Sridhar (2017) India [117]	MOPS > 4	Time from caudal block to post-operative rescue analgesia.	Yes	No	No
Srinivasan (2016) India [118]	VAS > 4	Time from caudal block to post-operative rescue analgesia.	Yes	Yes	No
Taheri (2010) Iran [119]	FLACC > 4	Time from caudal block to post-operative rescue analgesia.	Yes	No	Yes
Turan (2003) Turkey [120]	TPPPS > 3	Time from caudal block to post-operative rescue analgesia.	Yes	Yes	Yes
Vakkapatti (2019) India [121]	CHIPPS > 4	Not defined	Yes	No	No
Vetter^$ ^(2007) USA [122]	FLACC ≥ 4	Not defined	Yes	No	Yes
Weber (2003) Germany [123]	ObPS > 3	Not defined	Yes	No	No
Xiang (2013) China [124]	CHIPPS > 3	Not defined	No	No	Yes
Yao (2018) China [125]	CHIPPS ≥ 4	Time from caudal block to post-operative rescue analgesia.	Yes	No	No
Yildiz (2006) Turkey [126]	mCHEOPS > 5, VAS > 30 mm	Time from caudal block to post-operative rescue analgesia.	Yes	No	No
Yildiz (2010) Turkey [127]	CHIPPS ≥ 4	Time from caudal block to post-operative rescue analgesia.	Yes	No	No
Yousef (2014) Egypt [128]	CHEOPS & FLACC ≥ 4	Time from caudal block to post-operative rescue analgesia.	Yes	No	No

**Table 4:** Summary of characteristics. CHIPPS, Children & Infants Postoperative Pain; FLACC, Face, Legs, Activity, Crying & Consolability Scale; MOPS, Modified Objective Pain Scale ^a^n (%)

Characteristic	N = 89^a^	Characteristic	N = 89^a^
Year		Block guidance	
2010-2019	63 (71%)	Landmark	83 (93%)
2000-2009	24 (27%)	Ultrasound	3 (3.4%)
Before 2000	2 (2.2%)	USG	2 (2.2%)
Country		Not stated	1 (1.1%)
India	42 (47%)	Local anesthetic used	
Others	26 (29%)	Bupivacaine	58 (65%)
Egypt	11 (12%)	Ropivacaine	21 (24%)
Turkey	10 (11%)	Levobupivacaine	10 (11%)
Journal		Local anesthetic concentration	
Others	66 (74%)	0.25%	58 (65%)
Pediatric Anesthesia	9 (10%)	< 0.2%	30 (34%)
Indian Journal of Anesthesia	7 (7.9%)	0.50%	1 (1.1%)
Journal of Anesthesiology & Clinical Pharmacology	7 (7.9%)	Local anesthetic volume	
ASA Class		1 mL/kg	56 (63%)
I-II	62 (70%)	0.5 < conc < 1 mL/kg	13 (15%)
I	23 (26%)	0.5 mL/kg	12 (13%)
Not stated	3 (3.4%)	> 1 mL/kg	7 (7.9%)
I-III	1 (1.1%)	Not stated	1 (1.1%)
Age category		Pain scale	
Less than 14 years	52 (58%)	Others	42 (47%)
Less than 6 years	35 (39%)	FLACC	30 (34%)
Not stated	2 (2.2%)	CHIPPS	10 (11%)
Anesthesia type		MOPS	7 (7.9%)
General anesthesia	85 (96%)		
Sedation	4 (4.5%)		

Risk of Bias Assessments

For the primary outcome, duration of analgesia (n=87 RCTs), we adjudged 32 RCTs at low risk of bias, 48 RCTs with some concerns, and 7 RCTs at a high risk of bias. For the number of dose administrations (n=29 RCTs), we adjudged 11 RCTs at low risk of bias, 15 RCTs with some concerns, and 3 RCTs at a high risk of bias. For the number of dose administrations (n=18 RCTs), we adjudged 8 RCTs at low risk of bias, 6 RCTs with some concerns, and 4 RCTs at a high risk of bias. Inadequate details about randomization and allocation concealment were the most common reason for downgrading the rating, followed by concerns about outcome measurement. We have summarized these results in Table 5.

**Table 5:** Risk of bias assessments of included studies.

Author, Year, and Country	Randomization process	Deviations from intended interventions	Missing outcome data	Measurement of the outcome	Selection of the reported result	Overall bias
Abu-Elyazed (2017) Egypt [40]	Low	Low	Low	Low	Low	Low
Ahuja (2014) India [41]	Low	Low	Low	Low	Low	Low
Ahuja (2015) India [42]	Low	Low	Low	Low	Low	Low
Akin (2010) Turkey [43]	Low	Low	Low	Low	Low	Low
Al-Zaben (2015) Jordan [44]	Some concerns	Low	Low	Low	Low	Some concerns
Al-Zaben (2016) Jordan [45]	Some concerns	Low	Low	Low	Low	Some concerns
Aliena (2018) India [46]	Some concerns	Low	Low	Low	Low	Some concerns
Amitha (2019) India [47]	Some concerns	Some concerns	Low	Low	Low	High
Anand (2011) India [48]	Some concerns	Low	Low	Low	Low	Some concerns
Aziz (2016) Egypt [49]	Low	Low	Low	Some concerns	Low	Some concerns
Bhardwaj (2007) India [50]	Some concerns	Low	Low	Low	Low	Some concerns
Bonisson (2019) Brazil [51]	Some concerns	Low	Low	Some concerns	Low	High
Chertin (2016) Israel [52]	Some concerns	Low	Low	Some concerns	Low	High
Cho (2015) Republic of Korea [53]	Some concerns	Low	Low	Low	High	High
Choudhuri (2008) India [54]	Some concerns	Low	Low	Low	Low	Some concerns
Choudhary (2016) India [55]	Low	Low	Low	Low	Low	Low
Cook (1995) Scotland [56]	Some concerns	Low	Low	Low	Low	Some concerns
Dogra (2018) India [57]	Low	Low	Low	Low	Low	Low
El-Feky^#^ (2015) Egypt [58]	Low	Low	Low	Low	Low	Low
El-Hennawy (2009) Egypt [59]	Some concerns	Low	Low	Low	Low	Some concerns
El-Shamaa (2016) Egypt [60]	Low	Low	Low	Low	Low	Low
Farrag (2014) Egypt [61]	Some concerns	Low	Low	Low	Low	Some concerns
Fernandes^$ ^(2012) Brazil [62]	Low	Low	Low	Low	Low	Low
Gaitini (2000) Israel [63]	Some concerns	Low	Low	Low	Low	Some concerns
George (2018) India [64]	Low	Low	Low	Low	Low	Low
Goyal (2016) India [65]	Some concerns	Some concerns	Low	Low	Low	High
Gulec (1998) Turkey [66]	Some concerns	Low	Low	Low	Low	Some concerns
Gunes (2004) Turkey [67]	Some concerns	Low	Low	Low	Low	Some concerns
Gupta (2003) India [68]	Low	Low	Low	Low	Low	Low
Gupta (2009) India [69]	Low	Low	Low	Low	Low	Low
Gupta (2017) India [70]	Some concerns	Low	Low	Low	Low	Some concerns
Hegazy (2013) Egypt [71]	Low	Low	Low	Low	Low	Low
Jain (2018) India [72]	Low	Low	Low	Low	Low	Low
Joshi (2004) USA [73]	Some concerns	Low	Low	Low	Low	Some concerns
Kalsotra (2019) India [74]	Some concerns	Low	Low	Some concerns	Low	High
Kamal (2016) India [75]	Low	Low	Low	Low	Low	Low
Karaaslan (2009) Turkey [76]	Some concerns	Low	Low	Low	Low	Some concerns
Kaur (2016) India [77]	Some concerns	Low	Low	Low	Low	Some concerns
Khakurel (2018) Nepal [78]	Low	Low	Low	Low	Low	Low
Khatavkar (2016) India [79]	Low	Low	Low	Some concerns	Low	Some concerns
Kim (2014) South Korea [80]	Some concerns	Low	Low	Low	Low	Some concerns
Kim (2014) South Korea [81]	Some concerns	Low	Low	Low	Low	Some concerns
Koul (2009) India [82]	Some concerns	Low	Low	Low	Low	Some concerns
Kumar (2005) India [83]	Some concerns	Low	Low	Low	Low	Some concerns
Laha (2012) India [84]	Some concerns	Low	Low	Low	Low	Some concerns
Martindale (2004) UK [85]	Some concerns	Low	Low	Low	Low	Some concerns
Meenakshi Karuppiah (2016) India [86]	Low	Low	Low	Low	Low	Low
Memis (2003) Turkey [87]	Some concerns	Low	Low	Low	Low	Some concerns
Nafiu (2006) Ghana [88]	Low	Low	Low	Low	Low	Low
Narasimhamurthy (2016) India [89]	Low	Low	Low	Low	Low	Low
Neogi (2010) India [90]	Some concerns	Low	Low	Low	Low	Some concerns
Nisa (2019) Pakistan [91]	Some concerns	Some concerns	Low	Some concerns	Low	High
Odes (2010) Turkey [92]	Some concerns	Low	Low	Low	Low	Some concerns
Pan (2005) India [93]	Low	Low	Low	Low	Low	Low
Parameswari (2010) India [94]	Low	Low	Low	Low	Low	Low
Parameswari (2017) India [95]	Low	Low	Low	Low	Low	Low
Pathania (2003) India [96]	Some concerns	Low	Low	Low	Low	Some concerns
Paul (2010) India [97]	Some concerns	Low	Low	Low	Low	Some concerns
Potti (2017) India [98]	Low	Low	Low	Low	Low	Low
Prakash (2006) India [99]	Low	Low	Low	Low	Low	Low
Priolkar (2016) India [100]	Some concerns	Low	Low	Low	Low	Some concerns
Rawat (2019) India [101]	Low	Low	Low	Some concerns	Low	Some concerns
Ribeiro Jr (2011) Brazil [102]	Some concerns	Low	Low	Low	Low	Some concerns
Saadawy (2009) Egypt [103]	Some concerns	Low	Low	Low	Low	Some concerns
Sanwatsarkar (2017) India [104]	Low	Low	Low	Low	Low	Low
Sarvesh (2019) India [105]	Low	Low	Low	Some concerns	Low	Some concerns
Sayed (2018) Egypt [106]	Low	Low	Low	Some concerns	Low	Some concerns
Sayed (2018) Egypt [107]	Low	Low	Low	Some concerns	Low	Some concerns
Senel (2001) Turkey [108]	Low	Low	Low	Low	Low	Low
Sharpe (2001) UK [109]	Low	Low	Low	Low	Low	Low
She (2015) China [110]	Some concerns	Low	Low	Low	Low	Some concerns
Shirmohammadie (2019) Iran [111]	Low	Low	Low	Low	Low	Low
Shrestha (2010) Nepal [112]	Some concerns	Low	Low	Low	Low	Some concerns
Singh (2010) India [113]	Low	Low	Low	Low	Low	Low
Singh (2012) Nepal [114]	Some concerns	Low	Low	Low	Low	Some concerns
Sinha (2016) India [115]	Some concerns	Low	Low	Low	Low	Some concerns
Solanki (2016) India [116]	Some concerns	Low	Low	Low	Low	Some concerns
Sridhar (2017) India [117]	Some concerns	Low	Low	Low	Low	Some concerns
Srinivasan (2016) India [118]	Low	Low	Low	Low	Low	Low
Taheri (2010) Iran [119]	Some concerns	Low	Low	Low	Low	Some concerns
Turan (2003) Turkey [120]	Some concerns	Low	Low	Low	Low	Some concerns
Vakkapatti (2019) India [121]	Low	Low	Low	Some concerns	Low	Some concerns
Vetter^$^ (2007) USA [122]	Some concerns	Low	Low	Low	Low	Some concerns
Weber (2003) Germany [123]	Some concerns	Low	Low	Low	Low	Some concerns
Xiang (2013) China [124]	Some concerns	Low	Low	Low	Low	Some concerns
Yao (2018) China [125]	Low	Low	Low	Low	Low	Low
Yildiz (2006) Turkey [126]	Low	Low	Low	Low	Low	Low
Yildiz (2010) Turkey [127]	Some concerns	Low	Low	Low	Low	Some concerns
Yousef (2014) Egypt [128]	Some concerns	Low	Low	Low	Low	Some concerns

Results of Pairwise Meta-Analyses

All adjuvants significantly extended the analgesic duration compared to control except magnesium and morphine. All adjuvants except dexamethasone significantly reduced the number of doses required within 24 h. All adjuvants except clonidine reduced the total dose of acetaminophen needed within 24 h. These results were associated with significant heterogeneity (I2 > 50%), perhaps due to varying concentration and dosing of local anesthetic within studies. Formal publication bias assessment was not possible as many comparisons had fewer than 10 studies. Visual inspection of funnel plots did not suggest publication bias. We have summarized these results in the Section 3 in the Appendix.

Network Geometry

**Figure 2:**
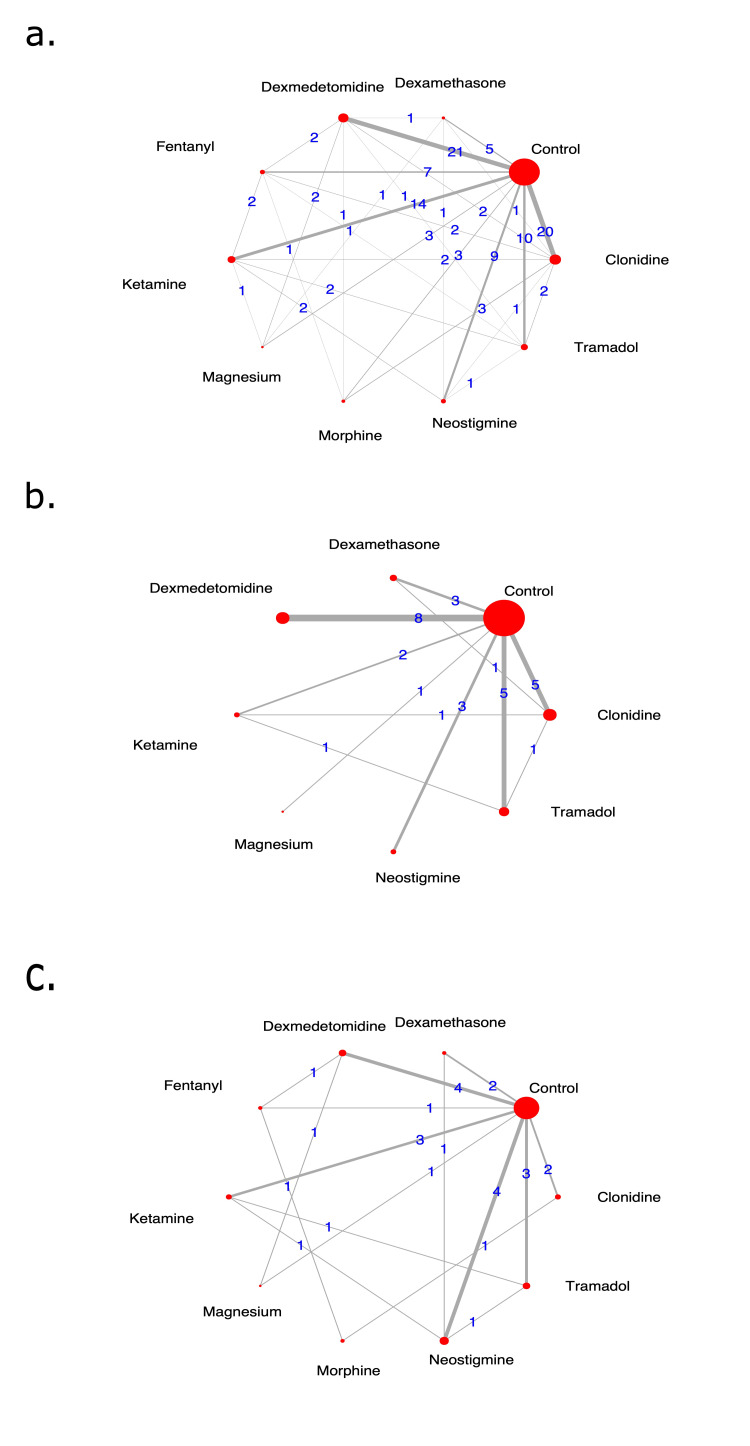
Network geometry for each outcome. The red circles represent interventions in each network, while a gray line connecting any work interventions represents a trial (or a trial arm in case of multi-arm studies). The total number of comparisons between any two interventions is printed as a number (in blue) on the respective gray line. Each intervention (red-circle) carries a label with its respective caudal adjuvant for each outcome. a. The network for primary outcome 'duration of analgesia' constituted 10 interventions and was assessed in 87 RCTs (n=5285 patients); b. The network for 'number of dose administrations' included eight interventions and was assessed in 29 RCTs (n=1765 patients), and c. The 'total dose of acetaminophen' network constituted ten interventions and was assessed in 18 RCTs (n=1156 patients).

We were able to assess all planned outcomes. The duration of the analgesia network constituted 10 interventions and was assessed in 87 RCTs (n=5285 patients). The most dominant nodes in this well-connected network were control (no adjuvant) vs. dexmedetomidine (n=21 RCTs), clonidine (n=20) and ketamine (n=14). The number of dose administrations network constituted eight interventions and was assessed in 29 RCTs (n=1765 patients). The most dominant nodes in this network were control (no adjuvant) vs dexmedetomidine (n=8 RCTs), clonidine (n=5), and tramadol (n=5). The total dose of the acetaminophen network constituted ten interventions and was assessed in 18 RCTs (n=1156 patients). The most dominant nodes in this network were control (no adjuvant) vs dexmedetomidine (n=4 RCTs), ketamine (n=3), and tramadol (n=3). These characteristics are shown in Figure 2.

Results of Network Meta-Analyses

Our analysis revealed that compared to control, neostigmine (WMD 513 min, 95% CI 402-625 min; n=9 RCTs, moderate certainty) prolonged the duration of analgesia the most, followed by tramadol (WMD 320 min, 95% CI 229-410 min; n=10 RCTs, low certainty) and dexmedetomidine (WMD 310 min, 95% CI 242-377; n=21 RCTs, low certainty). Based on an MCID of 100 min, morphine, magnesium, and fentanyl were not significantly better than control. Treatment rankings and SUCRA suggested that neostigmine was the best adjuvant, followed by tramadol and dexmedetomidine. 

Compared to control, dexmedetomidine was most effective at reducing the required number of dose administrations within 24 h (WMD - 1.2 dose, 95% CI - 1.6, -0.9 dose; n=8 RCTs, moderate certainty). This was followed by ketamine (WMD - 1.2 dose, 95% CI - 1.9, -0.5 dose; n=2 RCTs, low certainty) and tramadol (WMD - 1.1 dose, 95% CI -1.5, -0.7 dose; n=5 RCTs, very low certainty). Based on an MCID of 0.5 doses, clonidine, neostigmine, magnesium, and dexamethasone were not significantly better than control. Treatment rankings (SUCRA) suggested that dexmedetomidine was the best adjuvant, followed by ketamine and tramadol.

**Figure 3:**
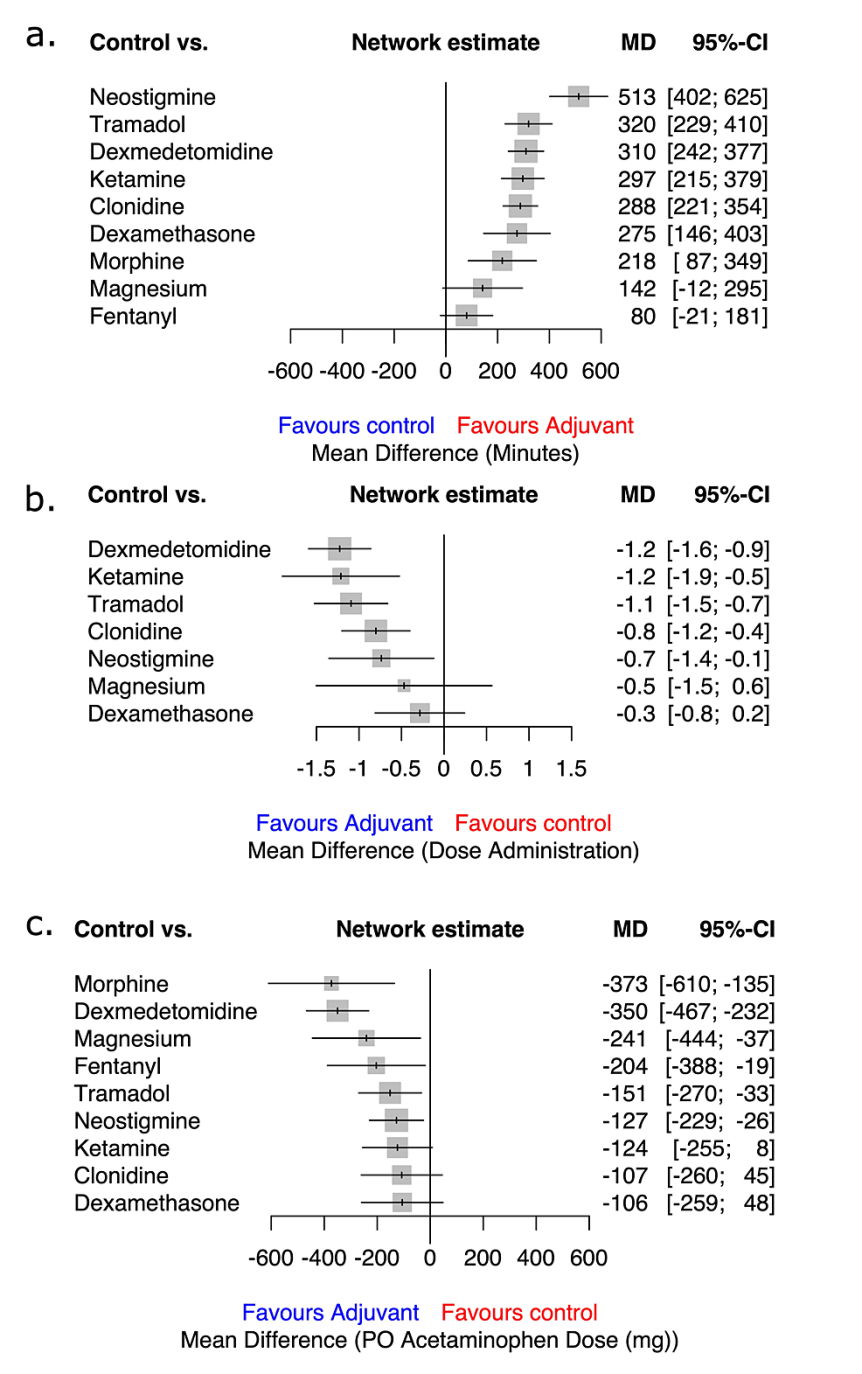
Forest plots included -- a. Duration of analgesia; b. The number of dose administrations; c. The total dose of acetaminophen. Each forest plot provides network estimates of included caudal adjuvants vs. control. A gray square represents the mean difference, while a black horizontal line represents the confidence interval. A vertical line represents the line of no effect. Units and values and the direction of the result are labeled below the x-axis for the respective outcome.

**Figure 4:**
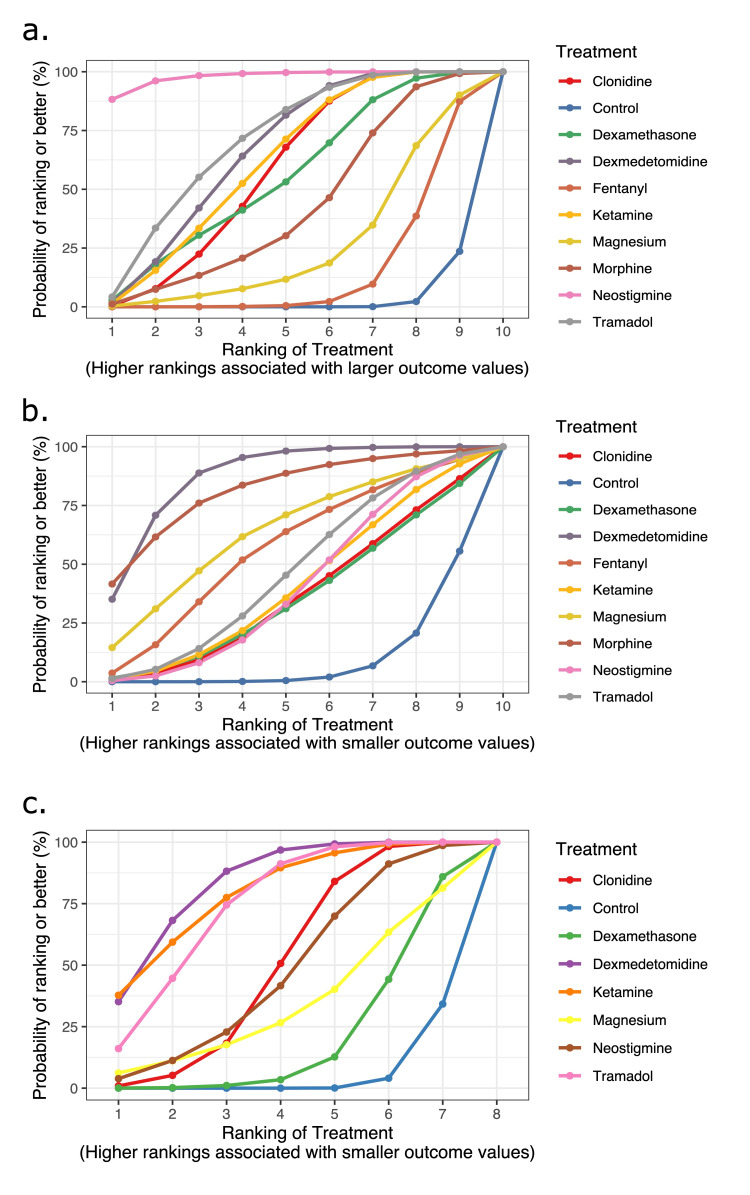
SUCRA (Surface Under the Cumulative Ranking curve) plots for outcomes -- a. Duration of analgesia; b. The number of dose administrations; c. The total dose of acetaminophen. The x-axis shows the possible ranks, and the y-axis the ranking probabilities. Each colored line connects the estimated probability of being at a particular rank for a caudal adjuvant. The area under the cumulative rankograms is between 0 and 100%. The larger the SUCRA, the higher the treatment in the hierarchy for an outcome.

Compared to control, dexmedetomidine was most effective at reducing the required number of doses within 24 h (WMD -350 mg, 95% CI -467, -232 mg, n=4 RCTs, moderate certainty). While morphine also reduced this dose (WMD -373 mg, 95% CI -610, -135 mg, moderate certainty), this evidence was an indirect comparison. Based on an MCID of 120 mg for acetaminophen use, no other adjuvant was superior to control. Treatment rankings (SUCRA) suggested that dexmedetomidine was the best adjuvant, followed by morphine. These results are depicted in Figure 3 (network plots) and Figure 4 (SUCRA plots) and summarized in Table 6 (net-league tables).

**Table 6:** Net-league tables for all outcomes. Treatments (or interventions) are reported in order of relative ranking for efficacy. Comparisons between treatments should be read from left to right. Their mean differences (and 95% confidence intervals) are in the cell in common between the column-defining treatment and the row-defining treatment. Mean differences above 0 favor the column-defining treatment for the network estimates and the row-defining treatment for the direct estimates.

Outcome 1. Duration of analgesia (minutes)									
Neostigmine	-199 (-629, 231)		483 (232, 733)	120 (-208, 448)	272 (-63, 607)				528 (405, 651)
194 (55, 332)	Tramadol	-126 (-455, 203)	69 (-181, 320)	283 (50, 516)				300 (-29, 629)	222 (110, 333)
204 (74, 333)	10 (-99, 119)	Dexmedetomidine		180 (-59, 418)	-44 (-372, 284)	444 (114, 774)	160 (-73, 392)	95 (-136, 326)	288 (215, 361)
216 (85, 347)	22 (-92, 137)	12 (-92, 117)	Ketamine	165 (-70, 400)			64 (-263, 391)	125 (-109, 359)	325 (232, 419)
225 (100, 351)	32 (-74, 137)	22 (-69, 112)	9 (-90, 109)	Clonidine	420 (89, 751)	56 (-140, 252)		342 (110, 574)	301 (225, 376)
239 (76, 401)	45 (-110, 200)	35 (-107, 176)	23 (-128, 173)	13 (-126, 152)	Dexamethasone		125 (-203, 453)		339 (191, 487)
295 (125, 466)	102 (-54, 258)	92 (-49, 233)	79 (-73, 231)	70 (-64, 204)	57 (-125, 238)	Morphine		90 (-238, 418)	356 (151, 562)
371 (184, 559)	178 (2, 354)	168 (7, 328)	156 (-10, 321)	146 (-19, 311)	133 (-57, 323)	76 (-124, 276)	Magnesium		103 (-89, 296)
433 (285, 581)	239 (112, 367)	229 (114, 344)	217 (95, 339)	208 (96, 320)	195 (33, 356)	138 (-15, 291)	62 (-120, 243)	Fentanyl	84 (-43, 211)
513 (402, 625)	320 (229, 410)	310 (242, 377)	297 (215, 379)	288 (221, 354)	275 (146, 403)	218 (87, 349)	142 (-12, 295)	80 (-21, 181)	Control
Outcome 2. Number of dose administrations									
Dexmedetomidine							-1.2 (-1.6, -0.9)		
-0.0 (-0.8, 0.8)	Ketamine	0.1 (-1.0, 1.2)	-0.6 (-1.9, 0.6)				-1.1 (-2.0, -0.3)		
-0.1 (-0.7, 0.4)	-0.1 (-0.9, 0.6)	Tramadol	-1.0 (-2.0, 0.0)				-0.9 (-1.4, -0.5)		
-0.4 (-1.0, 0.1)	-0.4 (-1.1, 0.3)	-0.3 (-0.8, 0.2)	Clonidine			-1.2 (-2.2, -0.2)	-0.8 (-1.3, -0.4)		
-0.5 (-1.2, 0.2)	-0.5 (-1.4, 0.4)	-0.4 (-1.1, 0.4)	-0.1 (-0.8, 0.7)	Neostigmine			-0.7 (-1.4, -0.1)		
-0.8 (-1.9, 0.3)	-0.7 (-2.0, 0.5)	-0.6 (-1.7, 0.5)	-0.3 (-1.4, 0.8)	-0.3 (-1.5, 0.9)	Magnesium		-0.5 (-1.5, 0.6)		
-0.9 (-1.6, -0.3)	-0.9 (-1.8, -0.1)	-0.8 (-1.5, -0.1)	-0.5 (-1.1, 0.1)	-0.5 (-1.3, 0.4)	-0.2 (-1.3, 1.0)	Dexamethasone	-0.5 (-1.1, 0.1)		
-1.2 (-1.6, -0.9)	-1.2 (-1.9, -0.5)	-1.1 (-1.5, -0.7)	-0.8 (-1.2, -0.4)	-0.7 (-1.4, -0.1)	-0.5 (-1.5, 0.6)	-0.3 (-0.8, 0.2)	Control		
Outcome 3. Total dose of acetaminophen (mg)									
Dexmedetomidine		-78 (-303, 147)	1 (-224, 226)						-352 (-470, -233)
23 (-226, 272)	Morphine		-184 (-417, 49)				-235 (-568, 97)		
-109 (-313, 95)	-132 (-438, 174)	Magnesium							-209 (-435, 16)
-146 (-337, 44)	-169 (-373, 35)	-37 (-301, 227)	Fentanyl						-77 (-302, 148)
-198 (-365, -32)	-221 (-486, 44)	-89 (-325, 146)	-52 (-271, 167)	Tramadol	-22 (-247, 203)	19 (-209, 247)			-160 (-297, -22)
-222 (-378, -67)	-245 (-504, 13)	-113 (-341, 114)	-76 (-287, 134)	-24 (-159, 111)	Neostigmine	-53 (-278, 172)		-87 (-317, 143)	-108 (-222, 6)
-226 (-402, -50)	-249 (-520, 22)	-117 (-359, 125)	-80 (-306, 147)	-28 (-183, 128)	-4 (-152, 145)	Ketamine			-114 (-256, 28)
-242 (-431, -54)	-265 (-506, -24)	-133 (-386, 120)	-96 (-315, 123)	-44 (-237, 149)	-20 (-203, 164)	-16 (-218, 185)	Clonidine		-100 (-262, 62)
-244 (-437, -51)	-267 (-550, 16)	-135 (-390, 120)	-98 (-338, 142)	-46 (-235, 143)	-22 (-189, 146)	-18 (-216, 180)	-2 (-218, 215)	Dexamethasone	-127 (-290, 36)
-350 (-467, -232)	-373 (-610, -135)	-241 (-444, -37)	-204 (-388, -19)	-151 (-270, -33)	-127 (-229, -26)	-124 (-255, 8)	-107 (-260, 45)	-106 (-259, 48)	Control

**Figure 5:**
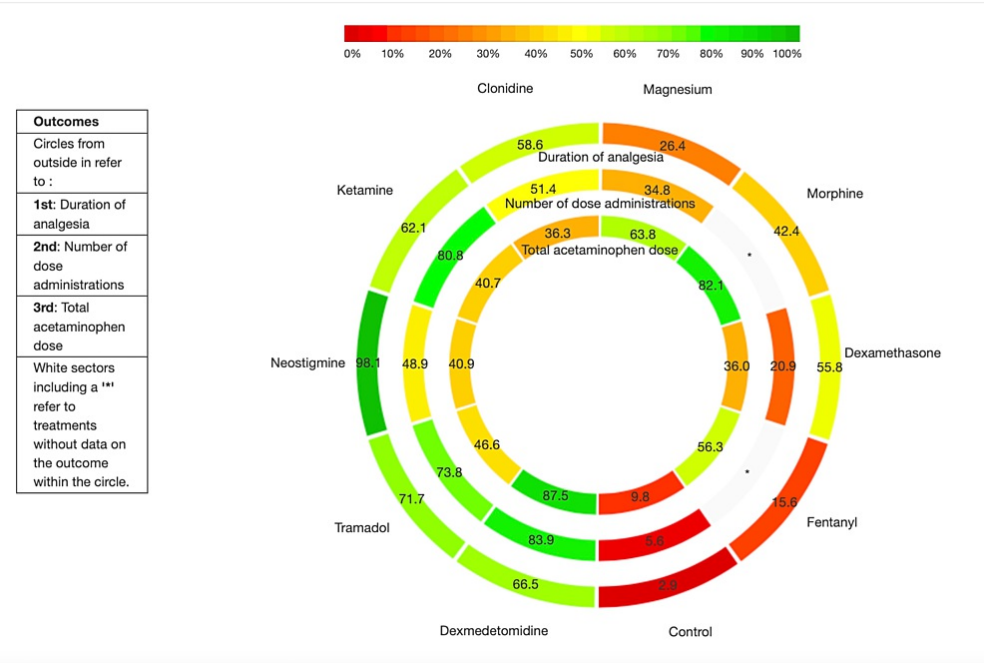
Rank heat plot. Each circle ring represents a different outcome, while each section represents a different treatment or intervention. Each sector is colored according to the ranking of the treatment at the corresponding outcome. The scale consists of the transformation of three colors (red, yellow, and green) and ranges from the lowest to the highest value of the ranking statistic, such as 0%-100% according to the ranking statistics (e.g., Surface Under the Cumulative Ranking curve [SUCRA]) values. The red color corresponds to the smallest ranking statistic value (0%), values near the middle of the scale are yellow, and the green color corresponds to the highest-ranking statistic value (100%). The rank heat plot analysis suggests that dexmedetomidine is the best overall adjuvant for all three outcomes, followed by Tramadol and Neostigmine. Fentanyl was the worst adjuvant.

We assessed all three outcomes using the rank heat-plot method described by Veroniki et al. [30]. Based on this, dexmedetomidine was judged to be the best adjuvant across all outcomes, followed by tramadol and neostigmine. Fentanyl fared worst among all adjuvants, while the control (no adjuvant) was the worst-ranking intervention. This is shown in Figure 5.

Inconsistency Assessment

We employed several methods to analyze inconsistency. We did not identify any evidence for global inconsistency for analgesia duration using frequentists and Bayesian methods. Exploration of local inconsistency using back-calculation methods revealed inconsistencies in clonidine vs. dexamethasone, clonidine vs. tramadol, dexmedetomidine vs. morphine, and neostigmine vs. tramadol comparisons. This was likely due to the paucity of direct trials in those comparisons. Given that there were only four comparisons among 30 for which direct evidence was unavailable, we concluded that the network for our primary outcome was consistent. 

We did not identify any evidence of global inconsistency for the number of dose administrations using frequentists and Bayesian methods. Exploration of local inconsistency using back-calculation methods reassured this conclusion. We did not identify any evidence for global inconsistency using frequentists and Bayesian methods for the total dose of the acetaminophen network. Node-splitting identified inconsistency in only dexmedetomidine vs. fentanyl comparison. Overall, we were assured of consistency in the network. These results are summarized in Table 7.

**Table 7:** Assessment of inconsistency. DIC, decision information criteria

Outcomes	Global consistency p-value from R (frequentist)	Global consistency p-value from STATA (frequentist)	Global consistency p-value from R (Bayesian)	Node-split analysis	Overall impression
Duration of analgesia	0.06	0.62	Consistency model (DIC 382) > Inconsistency model (DIC 384)	3 out of 30 comparisons are inconsistent	Consistency satisfied
Number of dose administrations	0.37	0.41	Consistency model (DIC 114) > Inconsistency model (DIC 115)	0 out of 11 comparisons are inconsistent	Consistency satisfied
Total dose of acetaminophen	0.40	0.96	Consistency model (DIC 82.6) > Inconsistency model (DIC 83)	1 out of 16 comparisons are inconsistent	Consistency satisfied

Risk of Bias Across Studies

**Figure 6:**
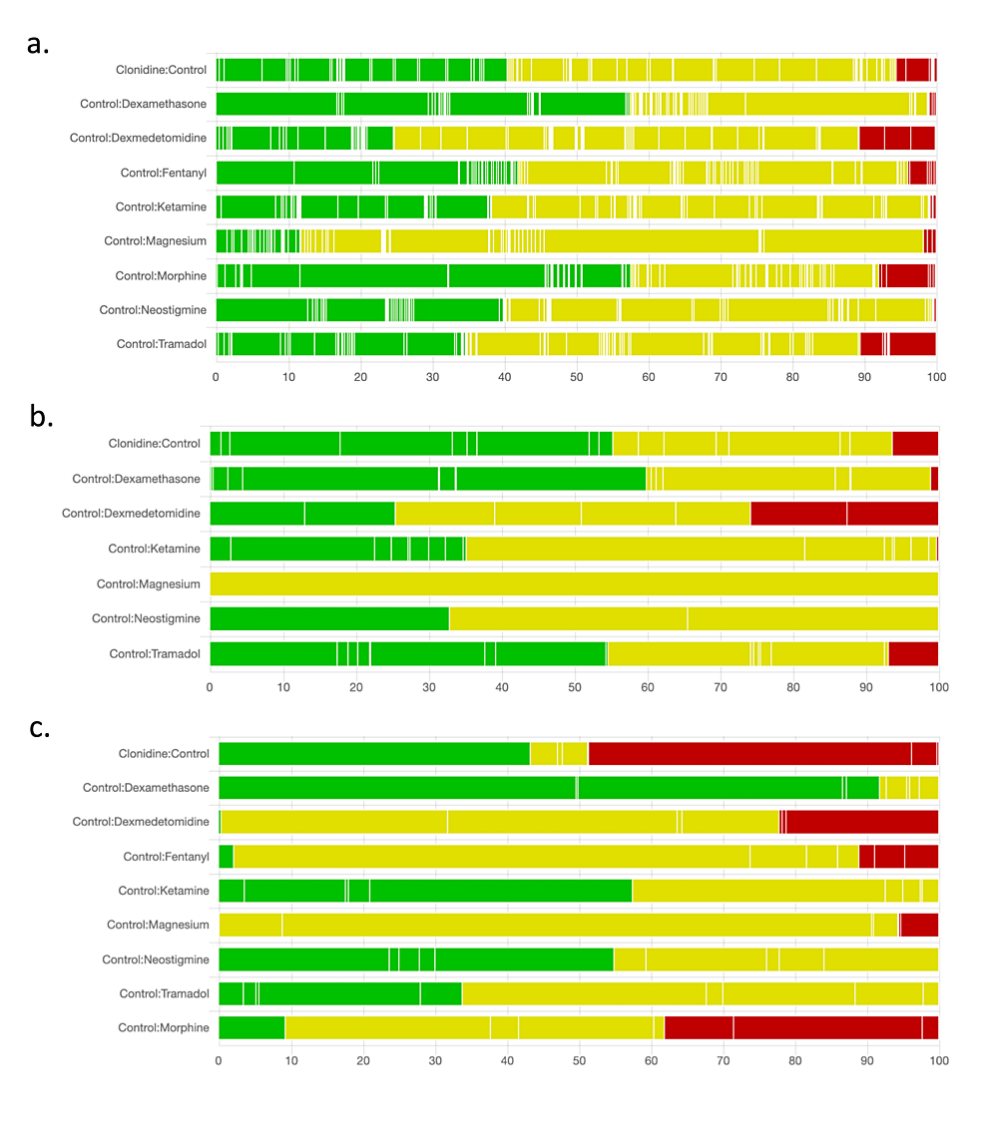
Comparison specific risk of bias for each outcome: a. duration of analgesia; b. number of dose administrations; and c. total dose of acetaminophen. Studies at low, unclear, and high risk of bias are depicted in green, yellow, and red color, respectively. Overall bias for each comparison is estimated by the majority rule.

**Figure 7:**
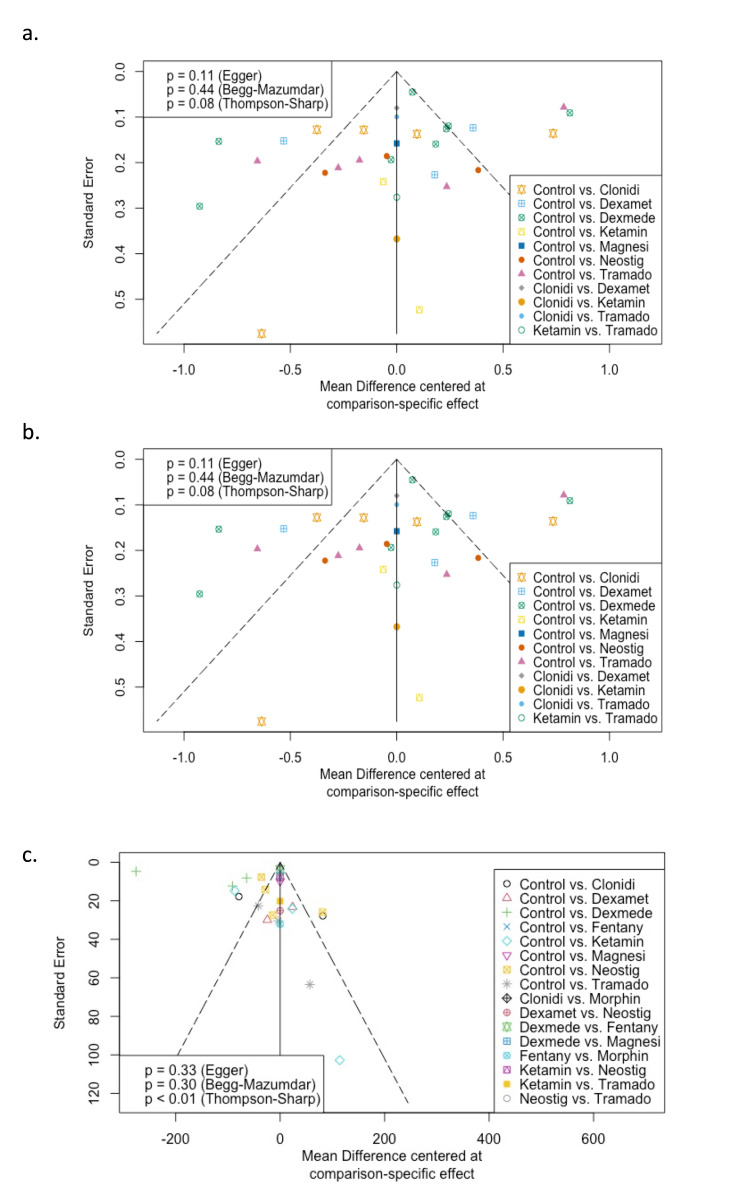
Comparison adjusted funnel plots for each outcome: a. duration of analgesia; b. number of dose administrations; and c. total dose of acetaminophen.

The proportion of direct evidence in each comparison loop was estimated using contribution matrices. Compared to control, network estimates for most adjuvants were predominantly informed by direct loops for all outcomes. The bias risk within each outcome's comparison loop was also assessed and used to inform certainty of evidence. Most loops were at some risk of bias, as shown in Figure 6. The comparison-adjusted funnel plot assessment did not yield any asymmetric plots, suggesting the absence of statistical evidence of publication bias. These results are shown in Figure 7.

Results of Additional Analysis

We assessed the impact of the inclusion of RCTs at high risk of bias (n=7 RCTs) using sensitivity analysis. The exclusion of these RCTs had no impact on the network estimates or the rankings of adjuvants. We also assessed the impact of volume-based dosing in caudal blocks in our studies through Bayesian network meta-regression. This confirmed that our findings were robust and not affected by variations in volume-based dose in RCTs included herein. Similarly, we did not identify any impact of the variation of concentration of local anesthetic used in the included RCTs on any outcome. We could not assess the impact of the type of local anesthetic and adjuvant dosing on outcomes due to resulting network disconnections and the fact that different adjuvants are used in different doses. 

Summary of Findings

Using the assessments above, we rated the certainty of the evidence for all analgesic outcomes. These results are shown in Table 8.

**Table 8:** Summary of findings. *NMA estimates are reported as weighted mean differences (WMDs) and 95% confidence intervals (CIs) as a frequentist model has been used. **Rank of treatment provides the comparative rankings of the treatment (best to worst) for a given outcome. The mean ranks and surface under the cumulative ranking curve (SUCRA) are also displayed. ***Indicated network meta-analysis estimates from indirect evidence only (no direct evidence available). Reasons for downgrading certainty assessment: 1 – Risk of bias; 2 – Heterogeneity; 3 – Inconsistency; 4 – Imprecision.

Comparison (vs. Control)	Number of RCTs	Number of patients	Direct evidence (%)	WMD (95%CI)*			Certainty of mixed evidence	Treatment rank (SUCRA)**
				Direct estimate	Indirect estimate	Network estimate		
Primary outcome: duration of analgesia (min)								
Neostigmine	9	420	82%	528 (405; 651)	447 (185; 708)	513 (402; 625)	Moderate^1^	1 (98)
Tramadol	10	520	66%	222 (110; 333)	509 (355; 664)	320 (229; 410)	Low^1,2^	2 (72)
Dexmedetomidine	21	1330	87%	288 (215; 361)	452 (265; 638)	310 (242; 377)	Low^1^	3 (67)
Ketamine	14	607	77%	325 (232; 419)	204 (-45; 463)	297 (215; 379)	Low^1,2^	4 (62)
Clonidine	20	960	77%	300 (225; 376)	246 (109; 383)	288 (221; 354)	Low^1,2^	5 (59)
Dexamethasone	5	462	75%	339 (191; 487)	81 (-175; 339)	275 (146; 403)	Very Low^2,3^	6 (56)
Morphine	3	130	41%	356 (151; 562)	123 (-48; 293)	218 (87; 349)	Very Low^2,3^	7 (42)
Magnesium	3	211	64%	103 (-89; 296)	209 (-45; 463)	142 (-12; 181)	Very Low^1,2,4^	8 (26)
Fentanyl	7	359	63%	84 (-43; 211)	74 (-91; 239)	80 (-21; 181)	Very Low^1,2,4^	9 (16)
Secondary outcome: Number of Dose Administrations (in doses, within 24-hours)								
Dexmedetomidine	8	501	100%	-1.2 (-1.6; -0.9)	-	-1.2 (-1.6; -0.9)	Moderate^1^	1 (84)
Ketamine	2	89	63%	-1.1 (-2.0; -0.3)	-1.3 (-2.5; -0.2)	-1.2 (-1.9; -0.5)	Low^1,2^	2 (81)
Tramadol	5	242	83%	-0.9 (-1.4; -0.5)	-1.9 (-3.0; -0.9)	-1.1 (-1.5; -0.7)	Very Low^1,2,3^	3 (74)
Clonidine	5	306	69%	-0.8 (-1.3; -0.4)	-0.7 (-1.4; 0)	-0.8 (-1.2; -0.4)	Moderate ^2^	4 (51)
Neostigmine	3	140	100%	-0.7 (-1.4; -0.1)	-	-0.7 (-1.4; -0.1)	Low^1,2^	5 (49)
Magnesium	1	77	100%	-0.5 (-1.5; -0.6)	-	-0.5 (-1.5; -0.6)	Very Low^1,4^	6 (35)
Dexamethasone	3	275	77%	-0.5 (-1.1; -0.1)	-0.5 (-0.6; 1.6)	-0.3 (-0.8; -0.2)	Very Low^2,3,4^	7 (21)
Secondary outcome: total acetaminophen dose (in mg, within 24 h)								
Dexmedetomidine	4	262	98%	-352 (-470; -233)	-255 (-1182; 731.38)	-373 (-610; -135)	Moderate^1^	1 (88)
Morphine***	-	-	-	-	-350 (-467; -232)	-350 (-467; -232)	Moderate^1^	2 (82)
Magnesium	1	60	82%	-209 (-435; 16)	-380 (-858; 96)	-241 (-444; -37)	Low^1,2^	3 (64)
Fentanyl	1	42	67%	-77 (-302; 148)	-464 (-787; -142)	-204 (-399; -19)	Very Low^1,2,3^	4 (56)
Tramadol	3	150	74%	-160 (-297; -22)	-128 (-359; 103)	-151 (-270; -33)	Very Low^1,2^	5 (47)
Neostigmine	4	194	79%	-108 (-222; 6)	-201 (-425; 24)	-127 (-229; -26)	Low^2^	6 (41)
Ketamine	3	129	85%	-114 (-256; 27)	-380 (-856; 96)	-124 (-255; 8)	Low^2,4^	7 (41)
Clonidine	2	110	89%	-100 (-262; 62)	-164 (-618; 291)	-107 (-260; 45)	Very Low^1,2,4^	8 (36)
Dexamethasone	2	200	89%	-127 (-290; 36)	64 (-394; 522)	-106 (-259; 48)	Low^2,4^	9 (36)

Discussion

Summary of Evidence

While previous attempts have been made to compare different adjuvants collectively [9, 12], our study is the first to perform a NMA and rank caudal adjuvants in order of their analgesic efficacy for all efficacy outcomes collectively. Based on the evidence from 89 RCTs (5442 patients), our study identified dexmedetomidine as the best caudal adjuvant across all analgesic outcomes (low to moderate evidence). On average, compared to using no adjuvant, dexmedetomidine prolonged the duration of analgesia by 310 min, reduced the number of analgesic dose administration by 1.2 doses, and reduced acetaminophen dose by 350 mg within 24 h of surgery. While other agents such as neostigmine or tramadol improve some outcomes, only dexmedetomidine consistently exceeded the pre-defined MCID thresholds for all outcomes.

Another fascinating insight from our results was that while tramadol and neostigmine prolonged the duration of analgesia (most likely by prolonging sensory block), they did not reduce the analgesic requirements. One explanation for this observation could be the lack of demonstrable synergism between epidural neostigmine [129] and systemic opioids, as opposed to epidural clonidine [130] and dexmedetomidine [131]. Similarly, epidural tramadol potentiates lidocaine-mediated sensory blocks in animal models [132]. Still, it is unknown if there is a synergism between caudal tramadol and systemic opioids. We observed that morphine and fentanyl reduced the need for acetaminophen dose despite not prolonging the analgesic duration. This likely points to the spinal and systemically mediated analgesic actions of these opioids [133] and differential spinal selectivity [134]. Even then, the evidence for morphine was predominantly indirect, while that for fentanyl was only marginally better than control. 

In contrast, caudal dexmedetomidine has been shown to mediate analgesia through local and systemic mechanisms. It binds to perineural post-synaptic a2 adrenergic receptors inhibiting synaptic transmission at pre-synaptic ganglionic sites; inhibits the release of substance P by stimulating a2 adrenergic receptors in substantia-gelatinosa of the dorsal horn, and prevents norepinephrine release at the dorsal horn [135-136]. Locally induced vasoconstriction also prolongs dexmedetomidine's locally mediated perineural effects [137]. Through systemic uptake, it binds to a2 adrenergic receptors producing centrally mediated analgesia, hypotension, bradycardia, and sedation [138-139]. However, its higher affinity to subtype 2A of a2 adrenergic receptors implies that its cardiovascular effects are less pronounced than non-selective agents such as clonidine [135, 140]. One beneficial impact of observed sedation is a reduced incidence of emergence delirium [8]. Given its local and systemic effects that aid analgesia, it is not surprising that our results confirm that dexmedetomidine consistently prolongs analgesia and reduces analgesic requirements. 

Several meta-analyses have compared the relative efficacy and adverse effects of various adjuvants such as alpha-2 agonists (clonidine [9] and dexmedetomidine [8]), N-methyl-D-aspartate (NMDA) agonists (ketamine [10] and magnesium [11]), opioids (fentanyl, morphine, and tramadol [12]), corticosteroids (dexamethasone [13-14]), and acetylcholine esterase inhibitors (neostigmine) [12]. However, such individual pairwise meta-analyses cannot provide all adjuvants' comparative effectiveness and relative rankings. This insight can only be obtained through an appropriately conducted NMA wherein multiple adjuvants can be assessed simultaneously, and both direct and indirect comparisons inform the mixed estimates. Indeed, our review is the first to report these estimates using a robust NMA analysis and interpretation. 

Using all adjuvants for neuraxial blocks (except epinephrine) remains an off-label indication. None of the included studies in our review evaluated the long-term neurological safety of caudal adjuvants. Such effects are best ascertained by examination or a delayed (two-week) follow-up questionnaire to assess deficits. Unfortunately, a pediatric population hinders a reliable neurologic assessment. While available data from animal and human studies indicate the safety of most adjuvants [141-143], drawing firm conclusions will likely require robust data on neurological safety. It is unlikely that a large-sized RCT would be carried out to assess this; in its absence, we will have to rely upon animal data or observational evidence [144-145]. Therefore, our findings are limited to establishing the relative efficacy of caudal adjuvants rather than safety. 

Limitations and Strengths

Our NMA is subject to a few limitations. First, available RCTs involved diverse demographics and methods, including variations in age, gender, and the type of infra-inguinal surgery. We observed variations in local anesthetics’ type, dose, concentration, and adjuvant doses. We mitigated this by employing a priori subgroups and meta-regression to explore heterogeneity and downgraded the evidence where appropriate. We could not assess the impact of the type of local anesthetic and adjuvant dosing on outcomes due to resulting network disconnections. Second, we observed some local inconsistencies attributed to design-by-treatment interactions (e.g., two-arm vs. three-arm trial) or a lack of an adequate number of trials. Third, some underlying biases (e.g., randomization and allocation concealment) were inherent to the source trials, leading us to downgrade the evidence strengths. Fourth, most of our studies were relatively small (n < 100), raising the possibility of small-study effects, overestimating treatment effect sizes, and inflating heterogeneity. Fifth, variations in the definitions and outcomes assessment may have contributed to heterogeneity and impacted the similarity assumption. Sixth, while we assessed publication bias at two stages (pairwise comparisons followed by the network) and found no evidence of such a bias, we cannot rule out its existence or impact on the network. Seventh, we chose not to assess the adverse effect of individual adjuvants in this review. This was due to two reasons: in general, most RCTs show a very low incidence of most adverse effects; and such low rates of complications, when taken together in a NMA framework, yield imprecise estimates that lack the required certainty to make any actionable recommendations. Eighth, we acknowledge that SUCRA and rankings can lead to misleading interpretations. Readers should form conclusions based on the certainty of evidence rather than rankings alone. Finally, we acknowledge that the use of most adjuvants used for perineural blocks remains off-label use, and their neurological safety is not well established. 

Despite these limitations, our article has several strengths. This is the first successful application of network methodology to the domain of caudal block adjuvants. It is also by far the largest meta-analysis on the topic. The internal validity of this review is enhanced by restricting inclusion to homogenous studies of a caudal block using long-acting local anesthetic agents. Further methodological strengths include prospective registration, comprehensiveness of literature search, scrutiny of network validity, and appraisal of observed differences in a predefined clinically important difference. Finally, we used the risk of bias assessment tools and GRADE recommendations designed explicitly for NMAs.

## Conclusions

Our results indicate that compared to control, neostigmine (moderate certainty), tramadol (low certainty), and dexmedetomidine (low certainty) are the most effective caudal adjuvants to prolong the duration of analgesia. Dexmedetomidine (moderate certainty), ketamine (low certainty), and tramadol (very low certainty) reduce the recommended analgesic dose frequency. The dose of acetaminophen needed is reduced most by dexmedetomidine (moderate certainty) and morphine (moderate certainty). Caudal adjuvants constitute an off-label use, and further research to establish their safety is needed.

## Figures and Tables

**Figure 8:**
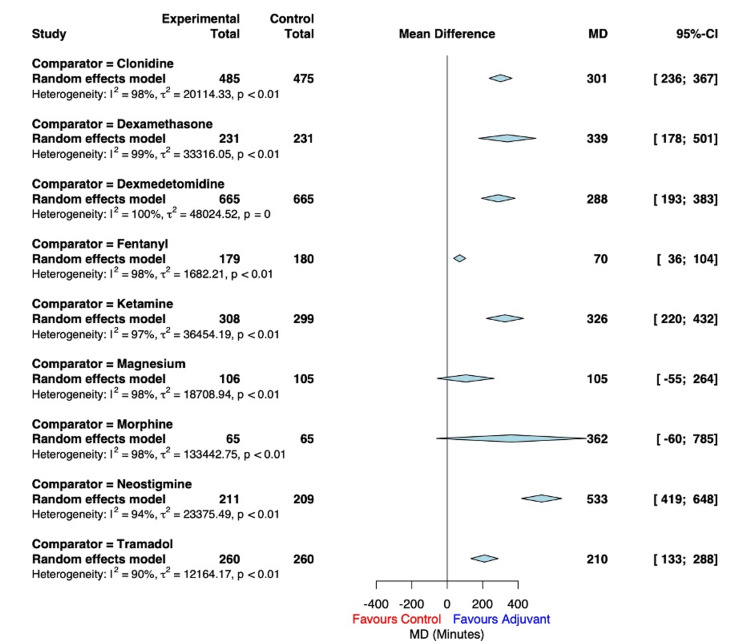
Forest plot showing pairwise analysis for each adjuvant vs. control for the duration of analgesia.

**Figure 9:**
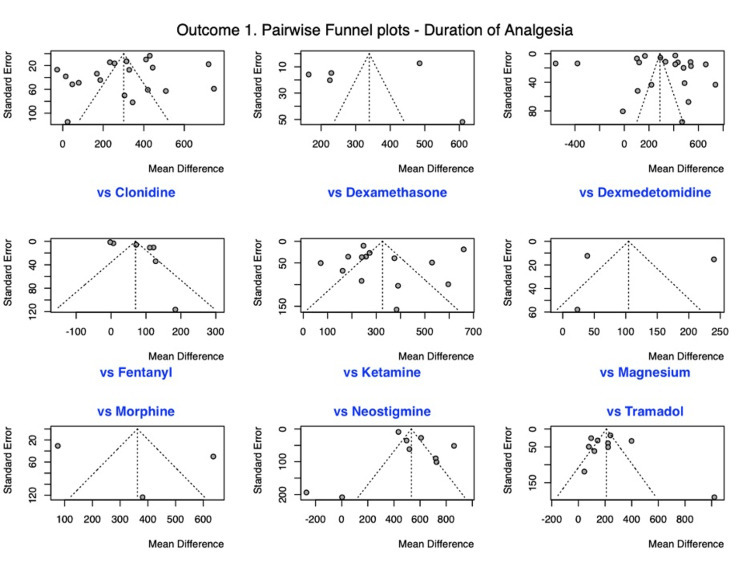
Publication bias assessment for all pairwise comparisons of caudal adjuvants vs. control for the duration of analgesia.

**Figure 10:**
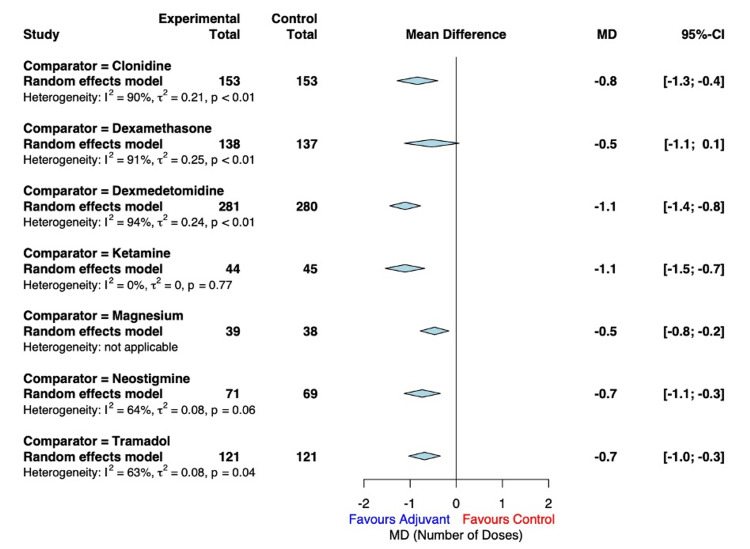
Forest plot showing pairwise analysis for each adjuvant vs. control for the number of dose administrations.

**Figure 11:**
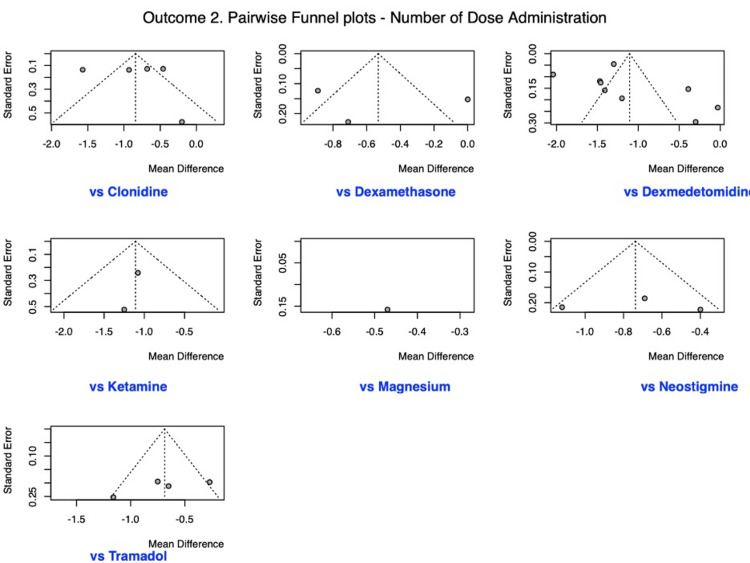
Publication bias assessment for all pairwise comparisons of caudal adjuvants vs. control for the number of dose administrations.

**Figure 12:**
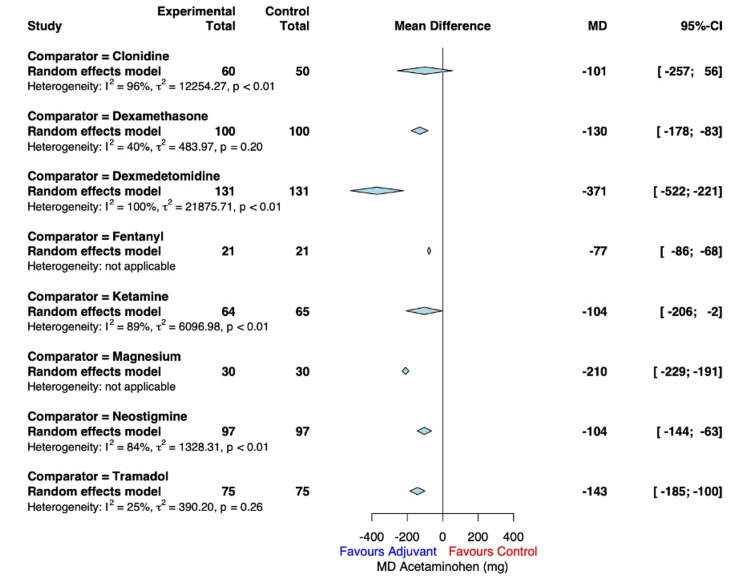
Forest plot showing pairwise analysis for each adjuvant vs. control for the total dose of acetaminophen.

**Figure 13:**
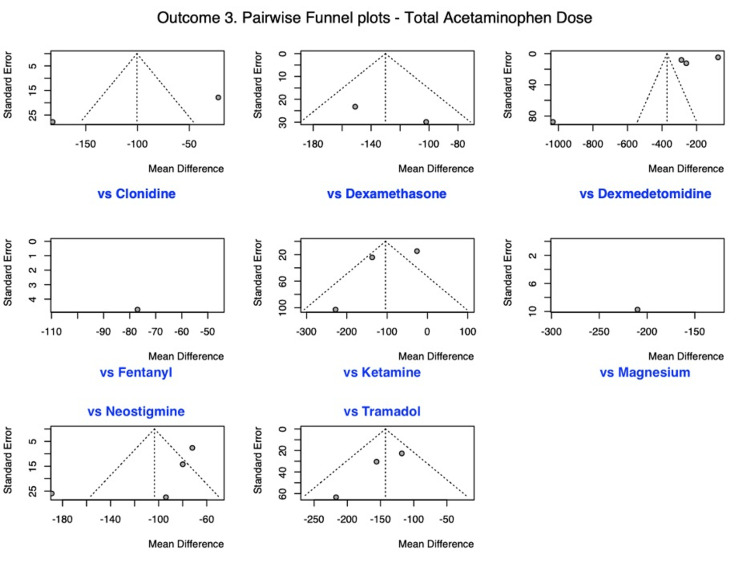
Publication bias assessment for all pairwise comparisons of caudal adjuvants vs. control for the total dose of acetaminophen.

## Appendices

Section 1. Protocol details

The protocol was prospectively registered on PROSPERO on 19 Sept 2018 (CRD42018108345). Link: https://www.crd.york.ac.uk/prospero/display_record.php?RecordID=108345.

There were no methodological amendments to the protocol once submitted.  The only deviation from protocol was the additional use of R software to generate other graphs and plots (using netmeta, gemtc, and BUGSnet packages). Besides this, we used STATA routines for NMA and CINEMA software to assess confidence in NMAs. We found this to be easier and automated in preference to the manual method suggested by the GRADE group. Both methods follow approximately the same methodology. 

Minimally clinical important differences were estimated as 25% of the average outcome estimate across the control group (mixed estimate) for each outcome. These were estimated to be:

Outcome

1. Duration of analgesia (average outcome value = 400 min); MCID = 100 min.

2. Number of dose administrations (average outcome value = 2 doses); MCID = 0.5 doses.

3. Total acetaminophen dose (average outcome value = 467 mg); MCID = 120 mg.        

Commands used

1. R Studio (netmeta package) - The main analysis of treatment effects, Network league tables, Global inconsistency testing - Wald test, Local Inconsistency testing - Node-splitting, Contribution matrix, Network funnel plots.

2. R Studio (BUGSnet package) - Bayesian - Network maps (better plots), Global inconsistency testing - Model fit (Consistency vs inconsistency; fixed vs random models), SUCRA, Network meta-regression (easier to perform; netmeta does not allow network meta-regression).

3. STATA (network package) - Global inconsistency testing - Wald test (occasionally, netmeta in R can give an error e.g., in case of zero or negative co-variances).

4. CINEMA - Risk of Bias across comparisons, Certainty of evidence.

All Bayesian models used the following parameters for MCMC chains: 

· n.adapt - number of adaptations for the mcmc chains = 1000

· n.burnin - number of burn-in iterations for the mcmc chains = 5000

· n.iter - number of iterations for the mcmc chains = 20000

· thin - thinning factor for the mcmc chains (default is 1) = 10

· n.chains - number of mcmc chains (default is 3) = 3

The adequacy of model parameters was tested using the Gelman-Rubin diagnostics, which should yield the ‘potential scale reduction factor' (PSRF) close to 1.

Section 2. Search strategy

We systematically searched the literature from three databases: MEDLINE, EMBASE, and PUBMED. There were no language restrictions imposed. The initial search was done on 26 May 2017 and revised on 30 June 2020.

Search strategy

Medline/ovid

1. *Anesthesia, Caudal/      

2. (caudal adj2 (anesthesia or anaesthesia or block)).ab,hw,kf,ot,ti  

3. 1 or 2         

4. (child$ or pediatric$ or infant$ or toddler$ or neonat$ or babies or baby).ab,hw,kf,ot,ti

5. exp Pediatrics/     

6. 4 or 5         

7. 3 and 6      

8. Urogenital surgical procedures/           

9. exp abdomen/su [surgery]        

10. (surg$ adj3 (abdominal or abdomen or urogenital or urologic$ or perineal)).ab,hw,kf,ot,ti.      

11. (hernia or inguinal or orchiopex$ or orchidopex$ or hydrocele or infraumbilic$ or infra-umbilic$).ab,hw,kf,ot,ti.      

12. 8 or 9 or 10 or 11           

13. 7 and 12  

14. (adjuvant$ or morphine or fentanyl or sufentanil or clonidine or bupivacaine or sevoflurane or tramadol or levobupivacaine or magnesium or neostigmine or ketamine or dexamethasone or dexmedetomidine).af        

15. 13 and 14

Embase

1. *caudal anesthesia/         

2. exp pediatrics/      

3. exp abdominal surgery/  

4. exp urologic surgery/      

5. (surg$ adj3 (abdominal or abdomen or urogenital or urologic$ or perineal)).ab,hw,kw,ot,sh,ti

6. (child$ or pediatric$ or infant$ or toddler$ or neonat$ or babies or baby).ab,hw,kw,ot,sh,ti     

7. (caudal adj2 (anesthesia or anaesthesia or block)).ab,hw,kw,ot,sh,ti       

8. 1 or 7         

9. 2 or 6         

10. 8 and 9    

11. (hernia or inguinal or orchiopex$ or orchidopex$ or hydrocele or infra-umbilic$ or infraumbilic$).ab,hw,kw,ot,sh,ti           

12. 3 or 4 or 5 or 11

13. 10 and 12            

14. (adjuvant$ or morphine or fentanyl or sufentanil or clonidine or bupivacaine or sevoflurane or tramadol or levobupivacaine or magnesium or neostigmine or ketamine or dexamethasone or dexmedetomidine).af        

15. 13 and 14

PubMed search strategy

(Anesthesia, Caudal[majr] OR caudal anesthesia[tiab] OR caudal anaesthesia[tiab] OR caudal block[tiab]) AND (child*[tiab] OR pediatric*[tiab] OR infant*[tiab] OR toddler*[tiab] OR neonat*[tiab] OR babies[tiab] OR baby[tiab] OR Pediatrics[mesh]) AND (Urogenital Surgical Procedures[mesh] OR Abdomen[mesh] OR abdominal surger*[tiab] OR urogenital surger*[tiab] OR urologic surger*[tiab] OR perineal surger*[tiab] OR hernia[tiab] OR inguinal[tiab] OR orchiopex*[tiab] OR orchidopex*[tiab] OR hydrocele[tiab] OR infraumbilic*[tiab] OR umbilic*[tiab]) AND (adjuvant*[all] OR morphine[all] OR fentanyl[all] OR sufentanil[all] OR clonidine[all] OR bupivacaine[all] OR sevoflurane[all] OR tramadol[all] OR levobupivacaine[all] OR magnesium[all] OR neostigmine[all] OR ketamine[all] OR dexamethasone[all] OR dexmedetomidine[all])

Section 3. Results of pairwise meta-analysis

Outcome 1. Duration of Analgesia

Forest plot (vs. Control) - direct comparisons only using Random-effects (DerSimonian and Laird) (please see Figure 8).

Assessment of publication bias (please see Figure 9).

Outcome 2. The Number of Dose Administrations.

Forest plot (vs. Control) - direct comparisons only using Random-effects (DerSimonian and Laird) (please see Figure 10).

Assessment of publication bias (please see Figure 11).

Outcome 3. Total Dose of Acetaminophen.

Forest plot (vs. Control) - direct comparisons only using Random effects (DerSimonian and Laird) (please see Figure 12).

Assessment of publication bias (please see Figure 13).

List of included studies

Eighty-nine RCTs were included in the NMA [40-128].  
